# A Highly-Conserved Single-Stranded DNA-Binding Protein in *Xanthomonas* Functions as a Harpin-Like Protein to Trigger Plant Immunity

**DOI:** 10.1371/journal.pone.0056240

**Published:** 2013-02-13

**Authors:** Yu-Rong Li, Wen-Xiu Ma, Yi-Zhou Che, Li-Fang Zou, Muhammad Zakria, Hua-Song Zou, Gong-You Chen

**Affiliations:** School of Agriculture and Biology, Shanghai Jiao Tong University, Key Laboratory of Urban (South) Ministry of Agriculture of China, Shanghai, China; Friedrich-Alexander-University Erlangen-Nurenberg, Germany

## Abstract

Harpins are produced by Gram-negative phytopathogenic bacteria and typically elicit hypersensitive response (HR) in non-host plants. The characterization of harpins in *Xanthomonas* species is largely unexplored. Here we demonstrate that *Xanthomonas* produce a highly conserved single-stranded DNA-binding protein (SSB_X_) that elicits HR in tobacco as by harpin Hpa1. SSB_X_, like Hpa1, is an acidic, glycine-rich, heat-stable protein that lacks cysteine residues. SSB_X_-triggered HR in tobacco, as by Hpa1, is characterized by the oxidative burst, the expression of HR markers (*HIN1*, *HSR203J*), pathogenesis-related genes, and callose deposition. Both SSB_X_- and Hpa1-induced HRs can be inhibited by general metabolism inhibitors actinomycin D, cycloheximide, and lanthanum chloride. Furthermore, those HRs activate the expression of *BAK1* and *BIK1* genes that are essential for induction of mitogen-activated protein kinase (MAPK) and salicylic acid pathways. Once applied to plants, SSB_X_ induces resistance to the fungal pathogen *Alternaria alternata* and enhances plant growth. When ssb_X_ *was deleted in* X. oryzae *pv.* oryzicola, *the causal agent of bacterial leaf streak in rice, the resulting* ssb_Xoc_ *mutant was reduced in virulence and bacterial growth* in planta, *but retained its ability to trigger HR in tobacco. Interestingly,* ssb_Xoc_ *contains an imperfect PIP-box (plant-inducible promoter) and the expression of* ssb_Xoc_ *is regulated by* HrpX, which belongs to the AraC family of transcriptional activators. Immunoblotting evidence showed that SSB_x_ secretion requires a functional type-III secretion system as Hpa1 does. This is the first report demonstrating that *Xanthomonas* produce a highly-conserved SSB_X_ that functions as a harpin-like protein for plant immunity.

## Introduction

Plants employ innate immune systems to overcome microbial pathogen infections [1], [2]. Pathogen-associated molecular patterns (PAMPs) comprise a diverse group of molecules such as flagellin [3], EF-Tu [4], chitin [5] and harpins [6]–[8]. PAMPs are known to elicit plant-triggered immunity (PTI); briefly, PAMPs are recognized by plasma membrane-localized receptor-like kinases (RLKs), which often contain nucleotide-binding domains and leucine-rich repeats [9]–[11]. Examples of RLKs include flagellin-sensitive 2 (FLS2) [12], the EF-Tu receptor EFR [13], and the chitin elicitor receptor kinase 1 (CERK1) [14]. These RLKs take similar roles to proteins encoded by plant resistance (*R*) genes for pathogen recognition [9], [10], [15], [16].

PTI is a form of basal defense or nonhost-mediated resistance in plants. PTI and effector-triggered immunity (ETI) activate similar signaling pathways and defense responses in plants. However, ETI generally activates a more prolonged, robust resistance than PTI [2]. Signal transduction pathways associated with PTI and ETI include mitogen-activated protein kinase (MAPK) cascades, calcium fluxes, and the activation of reactive oxygen species (ROS). Furthermore, both ETI and PTI are associated with modulations in hormonal signaling pathways including those associated with production of salicylic acid (SA) for systemic acquired resistance (SAR), jasmonic acid (JA) for induced systemic resistance (ISR) and ethylene (Eth) [17]. Unlike ETI, PTI-modulated signaling requires BAK1, which is a BRI1-ASSOCIATED KINASE 1 that regulates plant signaling by functioning as an adaptor for multiple RLKs [2], [17]–[20]. For example, the FLS2/BAK1 complex phosphorylates BIK1 (Botrytis-induced kinase 1) for signal transduction to the MAPK cascade [21]. The latter may then activate the expression of WRKY transcription factors that regulate SA-, JA- or Eth-dependent genes by binding the W-box [22]. However, it is unclear whether the proteins mentioned above are also involved in harpin-triggered immunity.

Recent studies have demonstrated that the α-helical structure of harpins is essential for HR induction, ion-mediated pore formation, development of curvilinear protofibrils or fibrils (amyloidogenesis), membrane-binding activities, ROS production and callose disposition [23]–[27]. Furthermore, multiple genes are activated in harpin-treated tobacco including those involved in hormone signaling [28], [29], HR markers (e.g. *HIN1* and *HSR203J*) [30], [31] and pathogenesis-related (*PR1a* and *PR1b*) [31]–[34]. Multiple reports document that harpin application promotes plant growth and induces SAR and ISR both to plant pathogens [28], [34] and insects [29], [35]. However, no reports have shown that harpin-elicited HR has any association with BAK1 in PTI-mediated signaling pathways.

Although the elicitation of HR in resistant host plants is commonly associated with ETI, it also occurs during PTI [36]. Harpins, which are glycine-rich, heat-stable proteins produced by the type-III secretion system (T3SS), are PAMPs that elicit HR and PTI [27], [37]. The first harpin described was HrpN, which is produced by the fire blight pathogen, *Erwinia amylovora* [7]. Multiple harpins can exist in a single phytopathogenic species; for example, *Pseudomonas syringae* pv. *tomato* DC3000 encodes four harpins, which are designated HrpZ1, HrpW1, HopAK1 and HopP1 [25]. In *Ralstonia solanacearum*, three harpins, PopA1 [38], HrpW [39], and PopW [40] have been identified. The HR elicited by harpins can be suppressed by eukaryotic metabolic inhibitors [6], [41], [42]. In *Xanthomonas*, the first harpin reported is HpaG in *X. axonopodis* pv. *glycines* [43], homologous to Hpa1 of *X. oryzae* pv. *oryzae* and *X. oryzae* pv. *oryzicola* and to XopA of *X. campestris* pv. *vesicatoria* [8], [43], but the latter does not elicit a HR in tobacco [43]. Interestingly, a *hpa1* deletion mutant still triggers a HR on nonhost tobacco [8], [44], [45], indicating that uncharacterized HR-elicitors are present in *X. oryzae* pv. *oryzicola*.

The genus *Xanthomonas* contains 307 species or pathovars that infect at least 124 monocotyledonous and 268 dicotyledonous plants and causes enormous agricultural losses [46]. Despite the huge host range of *Xanthomonas*, few species in this genus are known to cause disease on tobacco, suggesting that tobacco may sense a conserved molecule in *Xanthomonas* and potentially initiate plant immunity. In this report, we present evidence that a highly-conserved single-stranded DNA-binding protein (SSB_X_) in *Xanthomonas* is regulated by HrpX, secreted via the T3SS, required for full virulence *in planta*, and elicits HR in nonhost plants. These novel results indicate that SSB_X_ functions as a harpin-like protein and modulates plant immunity in tobacco.

## Materials and Methods

### Bacterial Strains and Growth Conditions

The bacterial strains and plasmids used in this study are listed in Table S1. The wild-type strains *X. oryzae* pv. *oryzicola* RS105, *X. oryzae* pv. *oryzae* PXO99^A^, *X. campestris* pv. *vesicatoria* 85–10, *X. axonopodis* pv. *citri* 306, *X. campestris* pv. *campestris* 8004, *R. solanacearum* ZJ3721 and *E. amylovora* 0065 (Table S1) were grown on nutrient agar (NA) or in nutrient broth (NB) [44] at 28°C. *Pst* DC3000 was grown on King’s Medium B [47]; *E. coli* and *A. tumefaciens* GV3101 were grown in Luria-Bertani (LB) medium [48] at 37°C and 28°C, respectively. *hrp*-inducing media included XOM3 for *X. oryzae* strains [49], XVM2 for *X. campestris* pv. *vesicatoria* 85-10 and *X. axonopodis* pv. *citri* 306 [50], and MMX for *X. campestris* pv. *campestris* 8004 [51]. MS medium was used for germination of plant seeds [52]. Antibiotics were used at the following concentrations (µg/ml): ampicillin (Ap), 100; kanamycin (Km), 50; rifampicin (Rif), 50; and spectinomycin (Sp), 100 µg/ml.

#### DNA manipulation

DNA isolations, subcloning, transformation, PCR, Northern blot and immunoblotting were conducted using standard procedures [53]. PCR primers are described in Table S2. PCR products were first cloned into pMD18-T (Takara, China) and then verified by sequencing. DNA sequences were analyzed with the VECTOR NTI software (http://www.invitrogen.com).

#### Determination of SSB_X_-elicited HR *in planta*

To investigate whether *ssb_Xoc_* triggers HR in tobacco, full-length *ssb_Xoc_* (552 bp) was amplified by PCR with the primer pairs *ssb_X_*-F/*ssb_X_*-R (Table S2) using the genomic DNA of strain RS105 as template. The amplified product was then cloned into PVX vector pgR107 [54] at *Cla*I and *Sal*I sites, resulting in pPVXssb_X_ (Table S1). The *hpa1* [8] and *bax* [55] genes were also cloned into pgR107, generating pPVXhpa1 and pPVXbax (Table S1), which were used as controls. These constructs (along with the empty vector) were transferred into *A. tumefaciens* GV3101, resulting in strains SSB_X_, Hpa1, Bax and PVX, respectively. Suspensions of *A. tumefaciens* strains were adjusted to OD_600_ = 0.5 and infiltrated into *N. benthamiana* with needleless syringes. HR symptoms were scored 48 hours post inoculation (hpi). Three independent experiments were performed and similar results were yielded. Representative results from one of these experiments are presented here.

### SSB Protein Expression and Purification

SSB_Xoc_ homologues were amplified from *X. oryzae* pv. *oryzicola* RS105, *X. oryzae* pv. *oryzae* PXO99^A^, *X. campestris* pv. *vesicatoria* 85-10, *X. axonopodis* pv. *citri* 306, *X. campestris* pv. *campestris* 8004, *R. solanacearum* ZJ3721 and *E. amylovora* 0065, *E. coli* BL21(DE3), *P. syringae* pv. *tomato* DC3000, and *P. fluorescens* (Table S1). Each ssb homologue was amplified by PCR from corresponding genomic DNAs using the primers listed in Table S2. PCR products were then cloned into pET41a (+) resulting in constructs designated pSSB*_Xoc_*_,_ pSSB*_Xoo_*, pSSB*_Xcv_*, pSSB*_Xac_*, pSSB*_Xcc_*, pSSB*_Rs_*, pSSB*_Ea_*, pSSB*_Ec_*, pSSB*_Pst_*, and pSSB*_Pf_* respectively (Table S1). These constructs were transformed into *E. coli* BL21 (DE3) (Table S1) as recommended in the Novagen pET System manual (Novagen, USA). Proteins were expressed as recommended by Novagen. Briefly, a single colony of each recombinant strain was inoculated to 2 ml LB broth containing Km. After incubation at 37°C for 12 h, 2 ml of culture was transferred into 200 ml of fresh LB liquid containing 1.0 mM isopropyl β-D-thiogalactopyranoside (IPTG, Sigma) and incubated for 4 h at 37°C. Cells were harvested by centrifugation, and pellets (1 g) were resuspended in 5 ml PBS buffer (137 mM NaCl, 2.7 mM KCl, 10 mM Na_2_HPO_4_ and 2 mM KH_2_PO_4_, pH 7.4); the solution also contained 20% glycerol, 5 U/ml DNaseI and 5 µl PMSF (phenylmethanesulfonylfluoride). Bacterial cells were lysed by sonication (20 kHz, 10 min). After centrifugation (15,000×*g*) for 15 min at 4°C, the supernatants were purified using a GSTrap™ FF column as recommended by the manufacturer (Purification Manual, GE Healthcare, Germany). The purified proteins were digested by thrombin to remove the GST-tag, and the purified proteins were quantified with the Easy Protein Quantitative Kit (TransGen Biotech, China) and a NANODROP 1000 Spectrophotometer (Thermo). Purified proteins were then used for HR induction assays in tobacco. The Hpa1 protein purified and the empty vector preparation (EVP) by the same procedure was used as a positive and negative control, respectively.

#### HR assays

Purified proteins were tested for their ability to elicit HR on *N. benthamiana* or *N. tabacum* cv. Xanthi by infiltration into plant tissues using needleless syringes. Plant responses were observed 48 hpi for the HR. All plants were grown in growth chambers at 25°C with a 12 h photoperiod. Experiments were repeated at least three times.

To measure minimal HR-eliciting concentrations, purified SSB_Xoc_ and others were diluted in PBS buffer at the following concentrations: 50, 25, 10, 5.0, 2.5, 1.0, 0.5, 0.1, 0.05 and 0.01 µM, while the purified Hpa1 diluted in PBS at the same concentrations above was used as positive control. All concentrations of the tested SSB proteins and Hpa1 were infiltrated into tobacco leaves and photographed 48 hpi.

To characterize biochemical activity, purified SSB_Xoc_ (1 µM) and Hpa1 (1 µM) were heat-treated at 100°C for 10 min and incubated with protease K (0.5 U/ml) at 37°C for 10 min, respectively. To investigate potential susceptibility to eukaryotic metabolic inhibitors, SSB_Xoc_ (1 µM) and Hpa1 (1 µM) were mixed with 1 µM LaCl_3_, 0.71 µM actinomycin D, and 0.1 µM cycloheximide, respectively. Treated and untreated SSB_Xoc_, Hpa1, and EVP were infiltrated into tobacco leaves. Three independent biological experiments were performed and yielded similar results. Representative results from one of these experiments are presented.

To determine whether the HR induced by SSB_Xoc_ was dependent on SA accumulation *in planta*, purified SSB_Xoc_ (1.0 and 5.0 µM) was infiltrated into wild-type and *NahG* tobacco, respectively. Purified Hpa1 (1.0 and 5.0 µM), wild-type strain RS105 (OD_600_ = 0.5), and EVP were used as controls. Three independent biological experiments were performed and yielded similar results. Representative results from one experiment are presented in this report.

#### DNA laddering assays

Genomic DNA of cv. Xanthi leaves infiltrated with purified SSB_Xoc_ (1 µM), Hpa1 (0.5 µM) and PBS buffer was isolated at 3, 6, 12, 24, 36 and 48 hpi, respectively. DNase-free RNase A was then used to digest existing RNA. Genomic DNA (10 µg) from each sample was subjected to electrophoresis in 2% agarose gels for at least 10 h under low voltage. Three independent experiments were performed and similar results were yielded. Representative results from one experiment are shown here.

#### H_2_O_2_ assays

Tobacco leaves (*N. benthamiana*) were infiltrated with purified SSB_Xoc_ (1 µM), Hpa1 (0.5 µM), and EVP, respectively, using needleless syringes. Eight hours later, treated leaves were collected and incubated in diaminobenzidine (DAB) for 8 h at 25°C, and then boiled in 95% ethanol for 10 min to remove the dye [56]. After 4 h further incubation in ethanol, leaves were fully bleached and brown precipitates were observed, indicating H_2_O_2_ accumulation and the production of ROS. Epidermal peels were performed at the injection sites 0 and 8 hpi with purified SSB_Xoc_ (1 µM), Hpa1 (0.5 µM), and EVP; these were then stained with DAB for 8 h at 25°C, and visualized with an OLYMPUS IX71 microscope. Three independent experiments were performed and similar results were yielded. Representative results from one experiment are presented here.

#### Callose deposition assays

To observe callose deposition, tobacco leaves (*N. benthamiana*) were infiltrated with purified SSB_Xoc_ (1 µM), Hpa1 (0.5 µM), or EVP with a needleless syringe. After infiltration (0 and 8 h), leaf epidermal peels of the infiltrated area were removed and incubated with aniline blue (0.1% in 0.15% K_2_HPO_4_, pH 8.2) for 0.5 h. Fluorescence (400 nm excitation and 510 nm emission) and bright-field images were obtained with an OLYMPUS IX71 microscope. Three independent experiments were performed and similar results were yielded. Representative results from one experiment are displayed here.

#### Northern blot assays

Total RNA of *N. benthamiana* leaves infiltrated with SSB_Xoc_ (1 µM), Hpa1 (0.5 µM) and EVP, was extracted 8 hpi. Prior to electrophoresis, RNA samples were treated with RNase-free DNaseI (TaKaRa, China) to remove potential traces of genomic DNA. RNA samples (30 µg) were then separated by electrophoresis in 1% agarose gels. Biotin-labeled DNA probes were prepared with the BrightStar Psoralen-Biotin Labeling kit (Ambion, USA) as recommended by the manufacturer. The primers for DNA probes are listed in Table S2. RNA was transferred to Hybond N+ membranes (Amersham Pharmacia Biotech, USA), hybridized with specific probes (Table S2) at 42°C using Northern Max (Ambion, USA), and detected using BrightStar BioDetect (Ambion) according to the manufacturer’s instruction.

### Assays for Plant Growth Promotion and Disease Prevention by SSB_Xoc_

To detect potential plant growth promoting activity of SSB_Xoc_, seeds of tobacco cv. Xanthi and *Arabidopsis. thaliana* (Col-0) were treated with SSB_Xoc_ (1 µM), Hpa1 (0.5 µM), EVP, and sterile water (DDW) for 8 h at 4°C. Treated seeds were placed on MS agar medium and measured for root length and fresh weight two weeks after treatment.

The potential effect of SSB_Xoc_ in enhancing plant disease resistance was investigated on tobacco inoculated with *A. alternata* strain TBA28A (Table S1), the causal agent of brown spot disease. Ten *plants of two-month-old tobacco were spray-inoculated with SSB_Xoc_ (1 *µM *in 0.5% Tween 20 solution*) *or Hpa1 (0.5 µM); plants were sprayed twice in three-day intervals. EVP was used as a negative control. Three days after the second spray, plants were inoculated with A. alternata* TBA28A *fresh disc. Infection was measured as the* diameter *of necrotic brown spots by* statistical analysis.

#### Construction of *ssb_Xoc_* deletion mutants

Experiments were designed to generate nonpolar deletion mutants of *ssb_X_* in *X. oryzae* pv. *oryzicola* RS105 and R*Δhpa1*, a *hpa1* deletion mutant [45]. The DNA sequences flanking *ssb_X_* were amplified from RS105 genomic DNA using primer pairs *ssb*I-F/*ssb*I-R and *ssb*II-F/*ssb*II-R (Table S2), cloned into pMD18-T (Takara, China), and verified by sequencing. After digestion with *Bam*HI/*Xho*I and *Xho*I/*Pst*I, the two fragments were cloned into the suicide vector pKMS1 [57] at *Bam*HIand *Pst*I sites, resulting in pKΔ*ssb_X_* (Table S1). This construct was introduced into the wild-type RS105 and the *hpa1* deletion mutant RΔ*hpa1*, and the isolation of *ssb_X_* deletion mutants was performed as described previously [57]. The *ssb_Xoc_* deletion mutant RΔ*ssb_X_* and the double mutant RΔ*hpa1*Δ*ssb_X_* were verified by PCR using primers *ssb*I-F/*ssb*II-R (Table S2) and by Southern blot analysis using *ssb_X_* as a probe.

#### Bacterial pathogenicity and HR assays

Pathogenicity assays were performed as described previously [8]. *X. oryzae* pv. *oryzicola* derivatives were examined for their ability to cause disease symptoms in rice IR24 (*Oryza sativa* ssp. *indica*) or to trigger a HR in tobacco cv. Xanthi. Rice adult plants (two-months-old) were inoculated by leaf-needling and fully-expanded tobacco leaves were infiltrated by needleless syringes with bacterial suspensions (∼3×10^8^ cfu/ml). Lesion lengths in rice were scored 14 days post-inoculation (dpi) and the HR in tobacco 2 dpi. All plants were maintained in growth chambers at 25°C with a 12 h photoperiod. Experiments were repeated at least three times.

#### Measurement of bacterial growth in rice

Bacterial cell suspensions (3×10^8^ cfu/ml; OD_600_ = 0.3) were infiltrated into recently expanded leaves of two-week-old rice IR24 with needleless syringes at three spots per leaf. Three 0.8 cm diameter leaf discs were harvested with a cork borer from each infiltrated area. The leaf discs were surface sterilized with 70% ethanol first and then with 30% hypochlorite, macerated with a sterile mortar and pestle in 1 ml of distilled water, serial dilutions were plated in triplicate on NA with appropriate antibiotics. Plates were incubated at 28°C for 3–4 days until single colonies could be counted. Bacterial numbers (cfu/cm^2^) were calculated, and standard deviations were determined using colony counts from three triplicate spots in each of three samples per time point per inoculum. Experiments were repeated at least three times.

#### Promoter activity assays and quantitative real-time PCR

To construct a transcriptional fusion between the *ssb_X_* promoter and glucuronidase (GUS), the promoter region (−1 to −350 bp) upstream of *ssb_X_* was amplified from the genomic DNA of *X. oryzae* pv. *oryzicola* RS105 with the primer pair pssb-F/pssb-R (Table S2). This PCR product was then fused with the promoter-less *gusA* gene, which was obtained with primers gusA-F/gusA-R (Table S2). The *ssb_X_*-*gusA* fusion was then cloned into pUFR034 [58] at the *Eco*RI site, resulting in pPIPAGUS (Table S1). In another experiment, a mutation was introduced into the PIP-box of the *ssb_X_* promoter using primers mpssb-F/pssb-R (Table S2) and fused with *gusA*, resulting in pPIPBGUS (Table S1).

For GUS activity assays, *X. oryzae* pv. *oryzicola* RS105 strain and *hrp* mutants were cultured in XOM3 to OD_600_ = 0.5. Bacterial cells were diluted and disrupted in sonication buffer (20 mM Tris-HCl, pH 7.0, 10 mM 2-mercaptoethanol, 5 mM EDTA, and 1% Triton X-100). GUS activities were determined every 30 min over a 3-h time period by measuring absorbance (*A*_415_) with p-nitrophenyl-D-glucuronide as the substrate [59]. One unit (U) was defined as 1 nmol of 4-methyl-umbelliferone produced per min per bacterium.

For quantitative real-time PCR analysis (qRT-PCR), the bacteria were cultured as described for the GUS activity assay in this report or cultured in rice suspension cells as described by Li and her colleagues [44]. Total RNA was extracted using Trizol reagent according to the manufacturer’s instructions (Invitrogen, USA). Total RNA was quantified by measuring the OD_260_/OD_280_, and the quality was examined by gel electrophoresis. Before synthesis of the first stranded, total RNA was treated with RNase-free DNaseI (TaKaRa, China) to remove genomic DNA. Removal of contaminating DNA was confirmed by using extracted RNA as a template to amplify selected target genes using the primers listed in Table S2. cDNA synthesis and PCR were conducted with AMV (TaKaRa) and *Ex*-Taq DNA polymerase (TaKaRa, China) using the primers listed in Table S2. Semi-quantitative RT-PCR was performed using the following program: step 1, 95°C for 3 min; step 2, 95°C for 20 s; step 3, 55°C for 30 s; step 4, 72°C for 40 s; 35 cycles of steps 2–4; and step 5, 72°C for 10 min. Quantitative real-time PCR was performed on an Applied Biosystems 7500 Real-Time thermocycler using SYBR *Premix Ex Taq*™ (Takara, China). Conditions for quantitative RT-PCR were as follows: denaturation at 95°C for 30 s and 41 cycles for 95°C, 5 s; 60°C, 34 s. The results were analyzed using Applied Biosystems 7300 System SDS software and the RQ study application. Expression of the *16S rRNA* gene was used as an internal standard to verify the absence of significant variation in cDNA levels. The comparative-threshold method by log_2_ value was used to calculate the relative mRNA level with respect to the corresponding transcript of *ssb_Xoc_* in the wild-type RS105 and the *hrpG* and *hrpX* mutants (Table S1) cultured in NA medium and rice suspension cells, respectively. All the real-time quantitative RT-PCRs were performed in triplicate.

#### SSB_Xoc_ secretion assays

To determine whether the secretion of SSB_Xoc_ was dependent on the T3SS, *X. oryzae* pv. *oryzicola* strains, containing *Ssb_Xoc_-c-myc* or *hpa1-c-myc* fusion (as a positive control) (Table S1), were pre-incubated in NB medium to logarithmic ***phase. Bacterial cells were harvested and*** adjusted to OD_600_ = 2.0 with sterilize water and washed twice. Then, 40 µl of bacterial suspension was poured into 1 ml of the *hrp*-inducing medium XOM3 [49] and incubated at 28°C for 8 h. Cell and supernatant fractions were separated by centrifugation, and the protein in the supernatant fraction was precipitated with 12.5% trichloroacetic acid. Proteins were separated on 10% sodium dodecyl sulfate-polyacrylamide gel electrophoresis (SDS-PAGE) gels and transferred to membranes for immunoblotting using anti-c-Myc primary antibody (Genescript, China). Primary antibodies were recognized by anti-rabbit secondary antibodies (Genescript, China) and visualized on autoradiographs with the Western-Light Chemiluminescence System (Transgene, Beijing, China). Three independent experiments were performed and yielded similar results. Representative results from one of these experiments are presented here.

## Results

### *ssb_Xoc_* Encodes a Single-stranded DNA-binding Protein Eliciting HR in Tobacco

**Figure 1:**
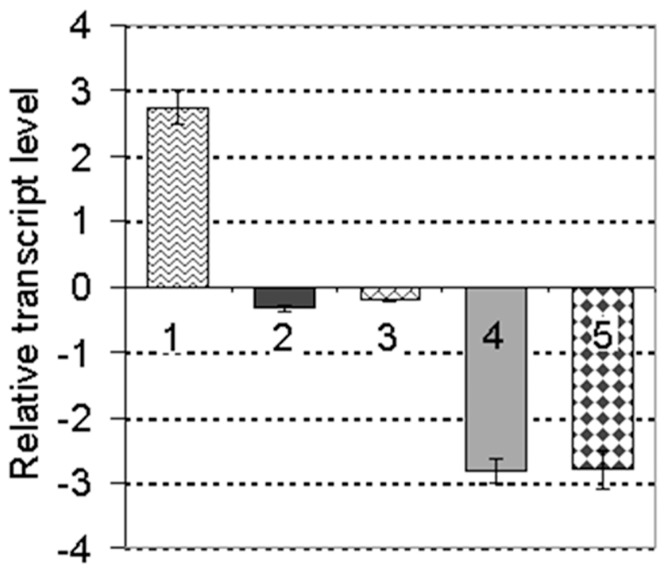
Expression of *ssb_Xoc_* is induced in rice suspension cells. Real-time quantitative PCR analysis of *ssb_Xoc_* transcript levels in *X. oryzae* pv. *oryzicola* wild-type RS105 and mutants RΔ*hrpG* and RΔ*hrpX*. Strains were grown in NB or rice suspension cells, and designated as (−) and (+), respectively. The ratios (shown in units of log_2_) reflect *ssb_Xoc_* transcript levels between different strains in two different growth conditions. 1. +RS105/−RS105; 2. −RΔ*hrpG*/−RS105; 3. −RΔ*hrpX*/−RS105; 4. +RΔ*hrpG*/+RS105; 5. +RΔ*hrpX*/+RS105. Data represent the means ± standard deviations (SD) from three replicates.

**Figure 2:**
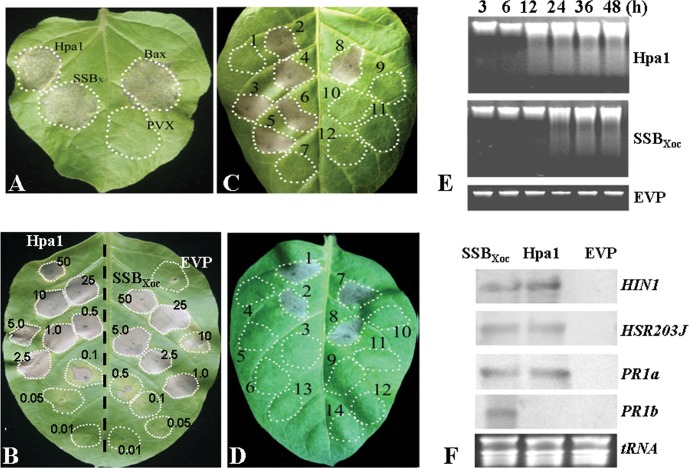
A highly conserved single-stranded DNA-binding protein (SSB) triggers a HR in tobacco. (A) HR induction by the SSB_Xoc_ protein of *X. oryzae* pv. *oryzicola*. *A. tumefaciens* GV3101 (OD_600_ = 0.5) containing *hpa1*, *ssb_X_* and *bax* genes in the PVX vector pgR107 was inoculated into *N. benthamiana* tobacco leaves with a needleless syringe. Hpa1 and Bax were used as positive controls, and *A. tumefaciens* containing the empty vector PVX was used as a negative control. (B) Concentration of SSB_Xoc_ required for HR induction in tobacco cv. Xanthi. Purified SSB_Xoc_ was diluted in PBS buffer and inoculated into tobacco with needleless syringes. Hpa1, which functions as a harpin of *X. oryzae* pv. *oryzicola*, was used as a positive control and EVP as a negative control. (C) HR assays in tobacco inoculated with SSB_X_ homologues obtained from various bacterial species. SSB proteins were overproduced in *E. coli*, purified (see Methods), and diluted in PBS buffer to different concentrations from 0.01 to 50 µM. A typical image of HRs on tobacco leaves caused by the proteins at 1 µM was taken in this report. Numbers represent sections of leaves inoculated with the following: 1, EVP; 2, SSB_Xoc_ from *X. oryzae* pv. *oryzicola* RS105; 3, SSB_Xoo_ from *X. oryzae* pv. *oryzae* PXO99^A^; 4, SSB_Xac_ from *X. axonopodis* pv. *citri* 306; 5, SSB_Xcv_ from *X. campestris* pv. *vesicatoria* 85-10; 6, SSB_Xcc_ from *X. campestris* pv. *campestris* 8004; 7, SSB_Pf_ from *P. fluorescens* Pf-5; 8, Hpa1_Xoc_, from *X. oryzae* pv. *oryzicola* RS105; 9, SSB_Ea_ from *E. amylovora* 0065; 10, SSB_Ec_ from *E. coli* BL21 (DES); 11, SSB_Rs_ from *R. solanacearum* ZJ2731; and 12, SSB_Pst_ from *P. syringae pv. tomato* DC3000. (D) Assays for SSBx and Hpa1-induced HR in response to various metabolic inhibitors. Tobacco plants were infiltrated with SSB_X_ (1 µM) or Hpa1 (0.5 µM), which was heat-treated or incubated (see methods) with one of the following: 1 µM LaCl_3_, 0.71 µM actinomycin D, 0.1 µM cycloheximide or protease K (0.5 U/ml). Leaf panels: 1, Hpa1; 2, heat-treated Hpa1; 3, protease K-treated Hpa1; 4, Hpa1 plus 1 µM LaCl_3_; 5, Hpa1 plus 0.71 µM actinomycin D; 6, Hpa1 plus 0.1 µM cycloheximide; 7, SSB_X_; 8, heat-treated SSB_Xoc_; 9, protease K-treated SSB_Xoc_; 10, SSB_Xoc_ plus LaCl_3_; 11, SSB_Xoc_ plus 0.1 µM actinomycin D; 12, SSB_Xoc_ plus cycloheximide; 13, distilled water; and 14, EVP. Leaves in panels A to D were photographed 24–48 h after infiltration. (E) Analysis of DNA laddering in SSB_Xoc_-treated tobacco leaves. Total genomic DNA was isolated from tobacco leaves 3, 6, 12, 24, 36 and 48 hpi with Hpa1 (0.5 µM), SSB_Xoc_ (1 µM) and EVP. DNA laddering was evaluated in 2% agarose gels. (F) Northern blot analysis in tobacco inoculated with SSB_X_, Hpa1, or EVP. The marker genes, *HIN1*, *HSR203J*, *PR1a* and *PR1b*, were chosen as the targets. Total RNAs were extracted from tobacco leaves infiltrated with SSB_Xoc_ (1 µM), Hpa1 (0.5 µM), or PBS buffer. Aliquots (10 µg each) of the extracted RNAs were separated in 1% agarose gels, transferred onto membranes, and analyzed by northern blotting. Blots were hybridized with digoxigenin-labeled *HIN1*, *HSR203J*, *PR1a* and *PR1b* cDNA. The experiment was conducted twice with similar results.

Mutagenesis of *hrpG* or *hrpX* in *X. oryzae* pv. *oryzicola* abolishes the elicitation of HR in tobacco and pathogenicity in rice [8]. Thus, we assumed that the expression of HR-eliciting genes, including *hpa1*, are also regulated by HrpG and HrpX [60]. Using cDNA microarrays of *X. oryzae* pv. *oryzicola* strain RS105 and the *hrpG* & *hrpX* mutants (unpublished), we discovered that the expression of *XOC_1514*, which encodes a single-stranded DNA-binding protein (AEQ95695.1) [61], was positively regulated by HrpG and HrpX in pathogen-infected rice cells (Fig. 1). This protein, which was designated SSB_Xoc_, is rich in glycine (20% of the total amino acids) but lacks cysteine residues (Table S3, Fig. S1); these are characteristics typical of the harpin protein family. To confirm this, we expressed *ssb_Xoc_* in PVX vector pgR107, which is typically used to screen HR elicitors in tobacco [54]. SSB_Xoc_ triggered HR in *N. benthamiana* that was similar to Hpa1 [8] and Bax [55] (Fig. 2A), suggesting that SSB_Xoc_ functions as a harpin in *X. oryzae* pv. *oryzicola.*

Previously, we reported that the minimum concentration of Hpa1 for HR induction is 0.1 µM [8]. To determine the concentration of SSB_Xoc_ required for HR induction, we over-expressed the protein in *E. coli* BL21 (DE3) (Table S1). Purified SSB_Xoc_ was infiltrated into tobacco at concentrations ranging from 0.01 to 50 µM. The minimum concentration of SSB_Xoc_ needed for HR induction in tobacco cv. Xanthi was 1.0 µM, approximately 10-fold higher than the minimum effective concentration of Hpa1 (Fig. 2B).

Nucleotide and protein searches using the NBCI database (http://blast.ncbi.nlm.nih.gov/Blast.cgi) indicate that SSB_Xoc_ homologues exist in other bacteria. Protein sequence alignment of SSB_Xoc_ with homologues from other Gram-negative bacteria indicated that the differences of SSB proteins between *Xanthomonas* and other prokaryotic bacteria mainly existed in the glycine-rich regions (see rectangle marked with dashes, Fig. S1). A phylogenetic analysis showed that SSB proteins could be classified into one of three groups (Fig. S2). Group I contained SSB proteins from closely related *Xanthomonas* species, group II SSB homologues from *Xyllela fastidiosa*, *R. solanacearum*, *Thermus aquaticus*, *P. aeruginosa*, and *P. syringae* pv. *tomato*, and group III from *Candidatus Liberibacter asiaticus*, *P. fluorescens*, *E. amylovora*, *Dickeya dadantii*, *Escherichia coli* and *Shigellia dysenteriae* (Fig. S2). Percentages of glycine-rich amino acids of SSB_X_ in *X. oryzae* pv. *oryzicola* RS105 strain and other bacteria are also shown in Table S3.

The bioinformatics analysis described above prompted us to investigate whether the SSB proteins from various bacterial species could elicit HR in tobacco. PCR was used to amplify *ssb* genes from *X. oryzae* pv. *oryzae* PXO99^A^, *X. campestris* pv. *campestris* 8004, *X. axonopodis* pv. *citri* (*Xac*) 306, *X. campestris* pv. *vesicatoria* 85-10, *R. solanacearum* ZJ3271, *P. syringae* pv. *tomato* DC3000, *P. fluorecens* Pf-5, *E. amylovora* 0065, and *E. coli* BL21 (DE3) (Table S1). *ssb* genes were amplified using the primers listed in Table S2, and then cloned into pET30a, generating pSSB constructs (Table S1) harbored by *E. coli* BL21 (DE3). The overproduced and purified SSB proteins at concentrations from 0.01 to 50 µM were infiltrated into tobacco cv. Xanthi with needleless syringes. Only did the SSB proteins from *Xanthomonas* elicited HR in tobacco, whereas those from other bacterial species did not ((Fig. 2C), suggesting that only SSB_X_ homologues, which are closely related and highly conserved in *Xanthomonas* (Fig. S1; Table S3), function as harpin.

Electrophoretic mobility shift assays (EMSA) demonstrated that SSB_Xoc_ from *X. oryzae* pv. *oryzicola*, as the representative of these proteins in *Xanthomonas* bound randomly synthesized single-stranded DNAs (DNA1 and DNA2, Fig. S3). This is consistent with that the single-stranded DNA-binding protein is for ssDNA protection from nucleolytic digestion in bacterial cell viability [62], implying that SSB_X_ of *Xanthomonas* rather than other plant pathogenic bacteria is coevolved to be recognized as a potential HR-elicitor by plants.

Previous reports indicate that harpin proteins are heat-stable and protease-sensitive [6], [7], [34], [41], [42]. To investigate these characteristic for SSB_X._, purified protein (1 µM) was incubated in a water bath at 100°C for 10 min and with protease K (0.5 U/ml) at 37°C for 10 min, while Hpa1 was used as positive control. Heat- or protease-treated SSB_Xoc_ was then inoculated into tobacco cv. Xanthi. At 48 hpi, heat-treated SSB_Xoc_ still triggered HR in tobacco (Fig. 2D panel 8), but protease-treated SSB_Xoc_ did not (Fig. 2D, panel 9).

### SSB_Xoc_-elicited HR is a form of Programmed Cell Death

The next experiments were designed to determine whether SSB_Xoc_, like Hpa1, is toxic to plant cells or not and SSB_Xoc_ leads to a metabolic response that triggers HR. Three metabolic inhibitors were used: actinomycin D (inhibits eukaryotic RNA polymerase II), cycloheximide (targets 80S ribosomes), and LaCl_3_ (a calcium channel blocker). These inhibitors were incubated with purified SSB_Xoc_ (see Methods) and then assayed for HR induction in tobacco. All three inhibitors prevented the SSB_Xoc_-elicited HR in tobacco when co-infiltrated with the purified SSB_Xoc_ (Fig. 2D, panels 12–14). These results indicated that the SSB_Xoc_-elicited HR is an active process and requires *de novo* gene expression, protein synthesis and calcium flux across membranes. Thus, SSB_Xoc_ acts as an elicitor, like Hpa1, of HR but is not directly toxic to plant cells.

It is well-documented that harpin-elicited HR is a form of programmed cell death (PCD), which is accompanied by DNA laddering [63]. To determine whether the SSB_Xoc_-elicited HR is a form of PCD, DNA laddering experiments were performed. Total genomic DNA from SSB_Xoc_-infiltrated tobacco leaves were extracted at different time points after infiltration and analyzed on 2% agarose gels. As shown in Fig. 2E, DNA ladders were clearly observed in SSB_Xoc_-inoculated leaves at 24 hpi, 12 h later than that in Hpa1-inoculated leaves. Thus, SSB_Xoc_, like Hpa1 (Fig. 2E), elicits PCD that is characterized by DNA laddering.

We then investigated whether SSB_Xoc_-elicited HR occurs with the activation of known HR marker genes including *HIN1* (harpin-induced 1) [64], *HSR203J* [65], and the SA-dependent marker, *PR1a* [66]; the JA-dependent gene, *PR1b* [67], was also conducted. The expression of these genes was evaluated in tobacco leaves infiltrated with SSB_Xoc_, Hpa1, and EVP 6 hpi. All four genes were induced in response to SSB_Xoc_; however, Hpa1 did not induce the expression of *PR1b* (Fig. 2F). Transcripts started to accumulate 6 hpi with SSB_Xoc_ and Hpa1 and were expressed at high levels up to 24 h (data not shown). These findings indicate that SSB_Xoc_-elicited HR was accompanied by the expression of HR markers and plant defense genes.

### SSB_Xoc_-elicited HR is Dependent on SA Accumulation

**Figure 3:**
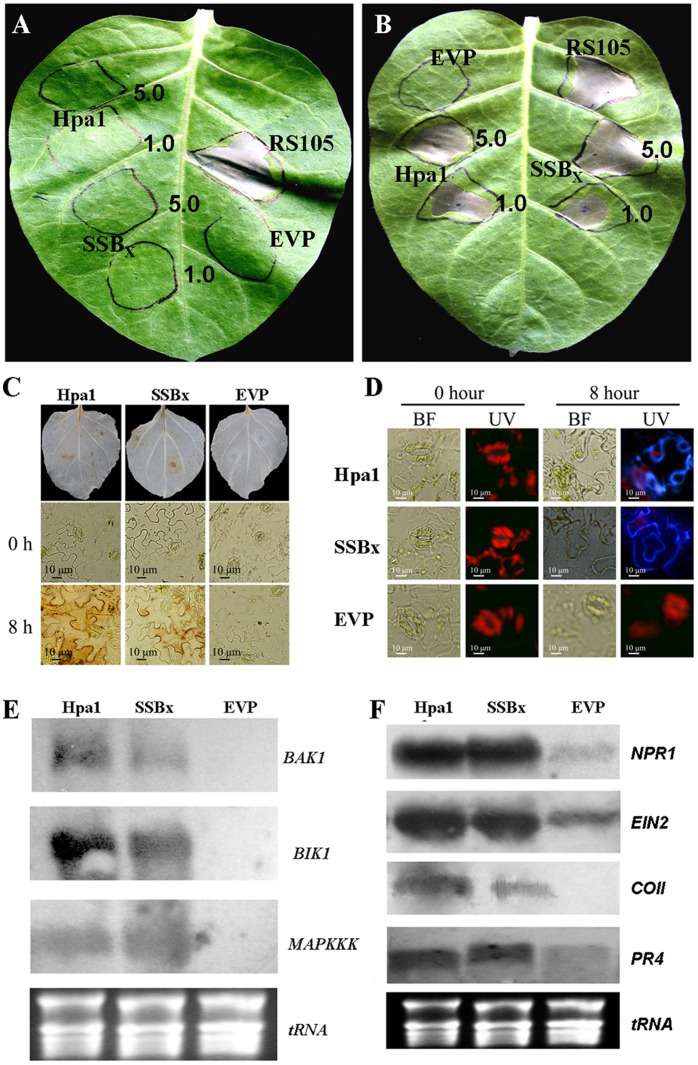
SSB_Xoc_ from *X. oryzae* pv. *oryzicola* may function as a PAMP and activates PTI in tobacco. SSB_Xoc_-triggered HR depends on the accumulation of salicylic acid (SA). *X. oryzae* pv. *oryzicola* strain RS105 (OD_600_ = 0.5), SSB_Xoc_ (1.0 and 5.0 µM), Hpa1 (1.0 and 5.0 µM), and EVP were inoculated into a *NahG* tobacco leaves (A) or wild-type tobacco cv. Xanthi (B). Photographs were taken 48 hpi. (C) SSB_Xoc_-triggered HR is accompanied by the oxidative burst. The production of H_2_O_2_ was evaluated in tobacco leaves by staining with 3,3′-diaminobenzidine tetrahydrochloride (DAB). The reaction mixture contained 200 ml of 0.5 mM DAB in 50 mM Tris acetate buffer (pH 6.0) with purified SSB_Xoc_ (1 µM) or Hpa1 (0.5 µM). Fully-expanded tobacco leaves were infiltrated with needleless syringe, incubated at room temperature for 0 and 8 h, and decolorized in 80% (v/v) ethanol for 10 min at 70°C. Leaves were examined with an OLYMPUS IX71 microscope. PBS buffer was included as a negative control. (D) SSB_Xoc_ elicits callose deposition in tobacco cell walls. Callose deposition in tobacco leaves was observed using fluorescence microscopy (OLYMPUS IX71) and staining with aniline blue at 0 and 8 hpi. Purified SSB_Xoc_ (1 µM) or Hpa1 (0.5 µM) was infiltrated into tobacco leaves (*N. benthamiana*) using needleless syringes, and EVP was used as a negative control. Inoculated epidermal peels were incubated with aniline blue for 0.5 h. Fluorescence images were captured using a 400 nm exposure for 510 nm absorbed light (UV), and bright-field (BF) images were captured using general bright light. Panels (E) and (F) show Northern blot analysis of PTI signaling pathways in tobacco treated with SSB_Xoc_, Hpa1, and PBS buffer (control). (E) The expression of *BAK1*, *BIK1* and *MAP3K*; (F) The expression of *NPR1*, *EIN2*, *COl1*, and *PR4*. Purified SSB_Xoc_ (1 µM) or Hpa1 (0.5 µM) was infiltrated into tobacco leaves using needleless syringe, and PBS buffer was used as a negative control. RNA was extracted 8 hpi and 10 µg aliquots were separated on 1% agarose gels and transferred to nylon membranes. Blots were hybridized with digoxigenin-labeled cDNA probes of the indicated genes.

It has been reported that HR induction by harpins requires SA accumulation [28], [68]. To investigate whether this is valid for SSB_Xoc_-elicited HR, we utilized transgenic tobacco expressing *NahG*; this line produces salicylate hydroxylase which degrades SA and blocks its accumulation [69]. Purified SSB_X_ and Hpa1 produced a HR in wild-type tobacco (Fig. 3B), but not in the *NahG* line (Fig. 3A). Thus, SSB_Xoc_-induced HR relies on SA accumulation *in planta,* which is the case for other harpins. It is noteworthy that the wild-type RS105 of *X. oryzae* pv. *oryzicola* still elicited HR in SA-deficient tobacco (Fig. 3A). Thus, in addition to SSB_Xoc_ and Hpa1, other unidentified HR-elicitor(s) exist(s) in *X. oryzae* pv. *oryzicola* to trigger HR on tobacco in SA-independence.

### SSB_Xoc_ Activates Plant Basal Defense

The oxidative burst, which involves the generation of ROS in response to microbial elicitors, occurs quite quickly in resistant plant cells [70], [71]. Thus we investigated whether the oxidative burst is generated in SSB_Xoc_-treated tobacco cells. At 8 hpi, DAB staining resulted in necrotic brown spots indicative of H_2_O_2_ production in both SSB_Xoc_- and Hpa1-infiltrated leaves (Fig. 3C).

Along with the oxidative burst, plants often mobilize multiple forms of basal defense to inhibit pathogen ingress, including callose deposition in cell walls [72]. To determine whether SSB_Xoc_ elicits callose deposition in tobacco, epidermal peels from SSB_Xoc_-infiltrated tissue were stained with aniline blue and examined by fluorescence microscopy. Both SSB_Xoc_- and Hpa1-infiltrated leaves showed evidence of callose deposition beginning at 8 hpi (Fig. 3D). Thus, SSB_Xoc_, like Hpa1, functions as an elicitor of basal defense responses and stimulates callose deposition.

The oxidative burst and callose deposition in tobacco infiltrated with SSB_Xoc_ prompted us to speculate that SSB_Xoc_ may function as a PAMP and activate the expression of genes involved in PTI signaling pathways. Our results indicate that *BAK1*, BIK1 and *MAP3K* (Fig. 3E), and *NPR1*, *EIN2*, *COl1* and *PR4* (Fig. 3F) genes are activated in response to SSB_Xoc_ or Hpa1. The data show that SSB_Xoc_ triggers a cascade of events similar to those triggered by Flg22 [12], [71], [73], [74], which leads to the oxidative burst and callose deposition and activates the expression of *PR* genes. These results support our presumption that SSB_Xoc_ acts as a PAMP like Hpa1.

### SSB_Xoc_ Induces Plant Disease Resistance and Promotes Plant Growth

**Figure 4:**
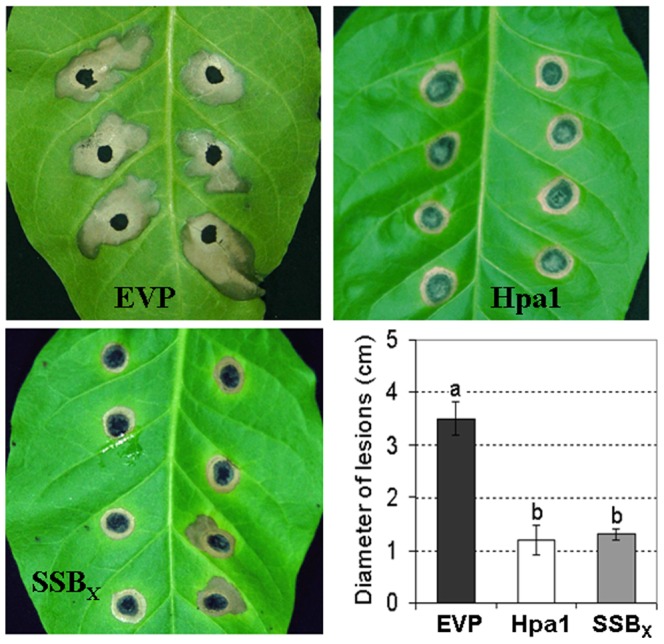
SSB_Xoc_ induces resistance to tobacco brown spot disease caused by *A. alternata*. Fully-expanded t*obacco leaves (cv. Xanthi) were sprayed twice in three-day intervals with purified SSB_Xoc_ (1 *µM), Hpa1 (*0.5 *µM) and EVP (negative control). *Three days after the second application of SSB_Xoc_, leaves were inoculated with A. alternata strain* TBA28A. *Diameters of brown spot lesions were measured and p*hotographed 14 dpi. Lesion size (diameter) are shown ± SD of triplicate measurements. Different letters above columns indicate significant differences at *P* = 0.01 using the Student’s *t* test.

Tobacco infiltrated with SSB_Xoc_ shows elevated expression of SA- and JA-dependent genes, along with the oxidative burst and callose deposition (Fig. 2F; Fig. 3C, D). Therefore, we hypothesized that SSB_Xoc_ may stimulate induced resistance to pathogen infection. For this, we inoculated a fungal pathogen, *A. alternata* TBA28A (Table S1), causal agent of tobacco brown spot disease, to fully-expanded tobacco leaves that were previously spray-inoculated twice with SSB_Xoc_ (1 µM) *in three-day intervals. The necrotic areas in tobacco leaves treated with SSB_Xoc_ were significantly smaller (*P* = 0.01,* t *test) than those observed on leaves inoculated with EVP (* *Fig. 4* *). Like* SSB_Xoc_, we found that *Hpa1 (*0.5 µM*) also induced similar resistance to A. alternata*. The data suggest that both SSB_X_ and Hpa1 induce SAR against pathogen infection.

**Figure 5:**
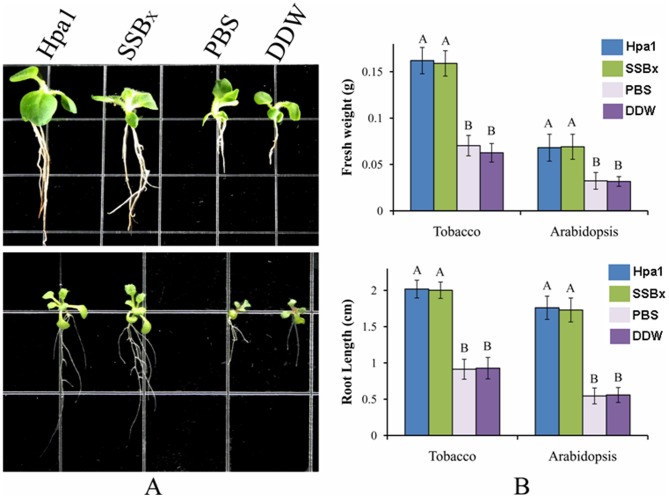
SSB_Xoc_ enhances plant growth. (A) Phenotype of tobacco (cv. Xanthi) and *Arabidopsis thaliana* (Col-0) grown on MS medium, 14 days after seed treatment with Hpa1 (0.5 µM), SSB_Xoc_ (1 µM), EVP, or double distilled water (DDW). Upper panel, tobacco; lower panel, *Arabidopsis*. (B) Fresh weight and root length of treated plants. Upper panel, fresh weight; lower panel, root length. Data are means ± SD of 50 randomly selected plants. Different letters above columns represent significant differences between treatments (*P* = 0.01 by *t* test).

The application of a harpin, HrpN from *E. amylovora*, enhances plant growth, particularly because the Eth-dependent genes are activated [30], [75]. The activation of Eth-dependent genes, e.g. *EIN2* and *PR4* (Fig. 3F), led us to determine whether SSB_Xoc_ promotes plant growth or not. For this, germinating seeds of *Arabidopsis thaliana* Col-0 and tobacco cv. Xanthi were soaked in a solution containing 1 µM SSB_Xoc_ for 8 h and then transferred to MS medium for 14 days. The results showed that root lengths of SSB_Xoc_-treated *Arabidopsis* and tobacco plants were nearly 2-fold longer than plants treated with EVP or water. There was no significant difference in root lengths between SSB_Xoc_ and Hpa1 treatments (*P = *0.01, *t* test; Fig. 5). In addition, fresh weight of SSB_Xoc_–treated plants, like those treated with Hpa1, was nearly three times more than those treated with EVP or water (Fig. 5B). Thus, SSB_Xoc_ promotes plant growth, possibly through the activation of the Eth signaling pathway (Fig. 3F).

### *ssb_Xoc_* is Required for Full Virulence and Bacterial Growth in Rice

**Figure 6:**
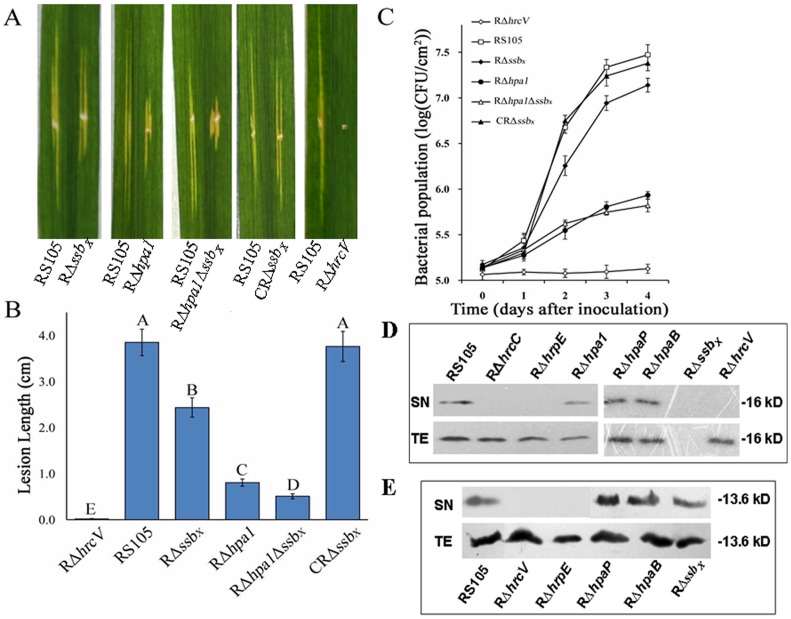
Secretion of SSB_Xoc_ depends on the T3SS and is required for full virulence and bacterial growth in rice. (A) Symptoms and (B) lesions lengths were used to assess the virulence of *X. oryzae* pv. *oryzicola* RS105 and selected mutants. One half of a rice leaf (IR24, two-months old) was inoculated with wild-type RS105, and the remaining half was inoculated with one of the following deletion mutants: *ssb_Xoc_* deletion mutant RΔ*ssb_X_*, *hpa1* mutant RΔ*hpa1*, the double mutant RΔ*ssb_X_*Δ*hpa1*, the complemented mutant CRΔ*ssb_X_*, and the T3SS mutant RΔ*hrcV*. Ten leaves were inoculated with each strain (OD_600_ = 0.3; approximately 3×10^8^ cfu/ml) by leaf-needling, and the assay was conducted in triplicate. Bacterial leaf streak symptoms were photographed 14 dpi, and representative symptoms are shown (A). The average lesion lengths formed by the wild-type and mutants were measured 14 dpi (B), and data represent means ± SD from three replicates. Different letters in each data column indicate significant differences at *P* = 0.01 (*t* test). (C) Bacterial growth assays *in planta*. Strains (OD_600_ = 0.3) were infiltrated into leaves of rice seedlings (IR24, two-weeks old) with blunt-end plastic syringes, and the cfu/cm^2^ of tissue was evaluated as described in Methods. Data represent means ± SD from three replications. (D) and (E) demonstrated the secretion of SSB_Xoc_ (D) and Hpa1 (E) are dependent on a functional T3SS of *X. oryzae* pv. *oryzicola*. This experiment utilized *X. oryzae* pv. *oryzicola* RS105 and strains containing mutations in the following genes: *hrcV* (RΔ*hrcV*), *hrcC* (RΔ*hrcC*), *hrpE* (RΔ*hrpE*), *hpaB* (RΔ*hpaB*), *hpaP* (RΔ*hpaP*), *hpa1* (RΔ*hpa1*) and *ssb_Xoc_* (RΔ*ssb_X_*) to express *ssb_Xoc_-c-myc* or *hpa1-c-myc* fusion (as a positive control). After incubation (8 h) in *hrp-*inducing medium XOM3, total cell extracts (TEs) and culture supernatants (SNs) were analyzed by SDS-PAGE and immunoblotted with an anti-c-Myc antibody. The immunoblotting assay was conducted twice, and similar results were obtained each time. For the detection of SSB_Xoc_, the strain RΔ*ssb_X_* with the empty vector pUFR034 was used as a negative control (D).

To investigate the potential contribution of SSB_X_ to virulence, *ssb_Xoc_* was deleted both in *X. oryzae* pv. *oryzicola* RS105 and the *hpa1* deletion mutant, RΔ*hpa1* (Table S1). The RΔ*ssb_X_*Δ*hpa1* double mutant (Table S1) was constructed using a two-step integration procedure [57]. Inoculation studies were conducted by inoculating one half of a rice leaf with wild-type RS105 and the remaining half with one of the following: *ssb_Xoc_* deletion mutant RΔ*ssb_X_*, *hpa1* mutant RΔ*hpa1* [44], the double mutant RΔ*ssb_X_*Δ*hpa1* (Table S1), the complemented strain CRΔ*ssb_X_* and the T3SS mutant RΔ*hrcV* [76]. Symptoms in RΔ*ssb_X_*-inoculated leaves were reduced relative to the wild-type strain, but were not as attenuated as RΔ*hpa1*-mediated symptoms (Fig. 6A). Lesion lengths in RΔ*ssb_X_*-inoculated leaves were significantly smaller than those induced by the wild-type RS105 (*P* = 0.01, *t* test) but larger than those induced by RΔ*hpa1* (Fig. 6B). The double mutant RΔ*ssb_X_*Δ*hpa1* did not lose pathogenicity in rice (Fig. 6A), but lesions were significantly smaller than those induced by the wild-type and single mutants (RΔ*ssb_X_* and RΔ*hpa1*) (Fig. 6B). As expected, the T3SS mutant, RΔ*hrcV*, produced no obvious disease symptoms in rice (Fig. 6A, B). Disease lesion lengths for the complemented strain CRΔ*ssb_X_* were equivalent to those induced by the wild-type RS105 (Fig. 6A, B), indicating that the mutant could be complemented for symptoms with the *ssb_Xoc_* gene *in trans*.

To determine whether *ssb_xoc_* contributes to growth of *X. oryzae* pv. *oryzicola* in rice, we compared the population dynamics of the wild-type RS105, RΔ*ssb_X_*, CRΔ*ssb_X_*, RΔ*hpa1*, RΔ*ssb_X_*Δ*hpa1*, and RΔ*hrcV*. The populations of RΔ*ssb_X_* were significantly lower than the wild-type RS105 beginning 2 dpi, but higher than the population of RΔ*hpa1* and RΔ*ssb_X_*Δ*hpa1* (Fig. 6C). Growth of RΔ*ssb_X_* was restored to wild-type levels when *ssb_Xoc_* was present *in trans* (Fig. 6C). These results indicated that *ssb_Xoc_*, like *hpa1*, contributes to bacterial growth *in planta*, although the effect was not as pronounced as seen with the T3SS mutant (RΔ*hrcV*) or RΔ*hpa1*. Furthermore, mutations in *ssb_Xoc_* and *hpa1* did not abolish the ability of the pathogen to elicit HR in tobacco (data not shown), implying that other HR-elicitor(s) exist in *X. oryzae* pv. *oryzicola*.

### SSB_X_ Secretion is Dependent on a Functional TTSS

The T3SS deficient mutant RΔ*hrcV* did not trigger HR in tobacco implies that HR elicitors, including SSB_Xoc_ and Hpa1, may be secreted via the T3SS. Bioinformatics analysis of SSB_Xoc_ did not show obvious T3SS secretion signals that are commonly found in T3SS effector proteins [77], [78]; thus it was not clear whether SSB_Xoc_ secretion required a functional T3SS. We used immunoblotting and SSB_Xoc_ tagged with a c-Myc epitope to explore whether SSB_xoc_ secretion was T3SS-dependent. The construct for expressing c-Myc-tagged SSB_Xoc_ was transferred into the wild-type and mutants defective in *hrcV* (encodes an inner membrane component of the T3SS), *hrcC* (encodes an outer membrane component), *hrpE* (encodes protein subunits of the Hrp pilus), *hpaB* and *hpaP* (encode exit control proteins for T3SE secretion) [8]. These mutants were designated RΔ*hrcV*, RΔ*hrcC*, RΔ*hrpE*, RΔ*hpaB* and RΔ*hpaP* (Table S1), respectively. RΔ*ssb_X_* with the empty vector pUFR034 was used as a negative control. When the wild-type RS105 and mutants RΔ*hrcC*, RΔ*hrcV*, RΔ*hrpE*, were incubated in a *hrp*-inducing medium XOM3 [50] and examined by immunoblotting, the SSB_Xoc_-c-Myc protein, like Hpa1-c-Myc protein (as a positive control), was found in the supernatants (SN) of the wild-type and RΔ*hrpE*, RΔ*hpaB,* and RΔ*hpaP* but absent from the SN fraction of RΔ*hrcV*, RΔ*hrcC* and RΔ*hrpE* (Fig. 6D, E). These results indicate that a functional T3SS is needed for secretion of SSB_Xoc_ and Hpa1. The SSB_Xoc_-c-Myc protein was detected in the SNs and total extracts (TE) of RΔhpaB and RΔhpaP, indicating that HpaB and HpaP are not required for secretion of SSB_Xoc_ and Hpa1 (Fig. 6D, E). Moreover, the secretion of Hpa1 was not impaired by the mutagenesis in *ssb_Xoc_*, and vice versa (Fig. 6D, E).

#### *ssb_X_* is positively regulated by HrpX

**Figure 7:**
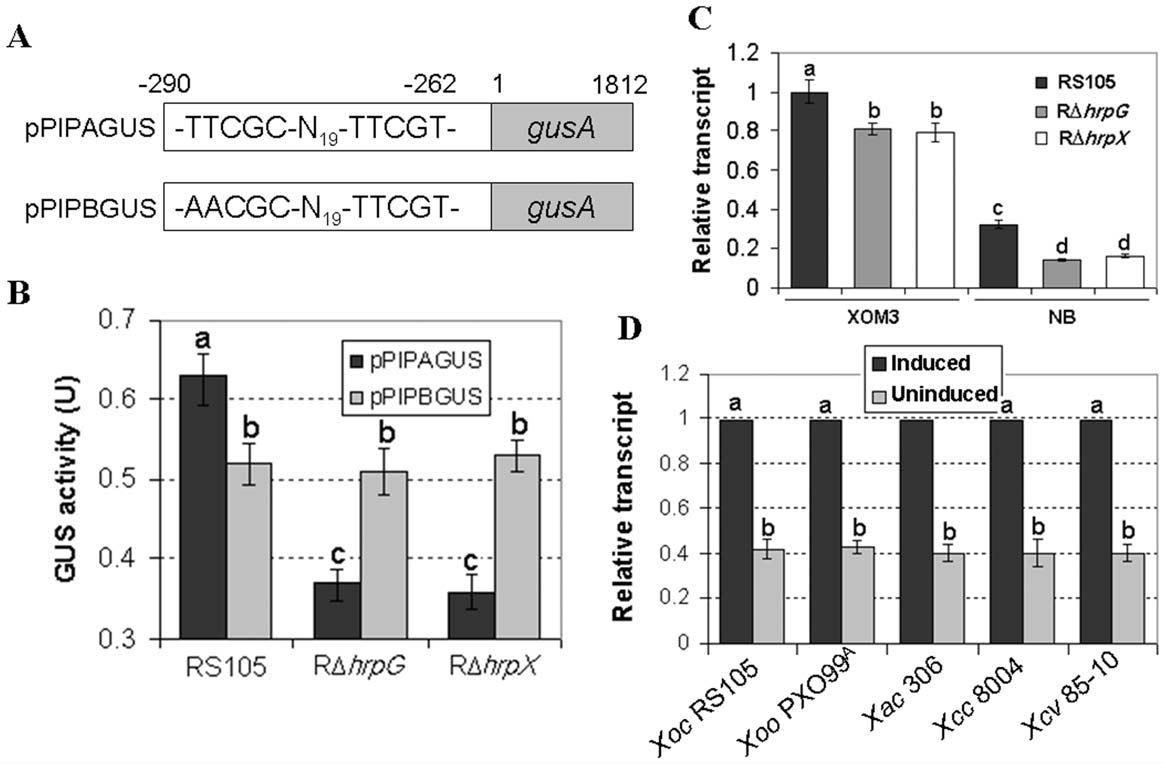
*ssb_X_* is expressed in *hrp*-inducing conditions and regulated by HrpG and HrpX. (A) Schematic map of a transcriptional fusion where the *ssb_Xoc_* promoter of *X. oryzae* pv. *oryzicola* RS105 is fused to the *gusA* reporter gene. Upper panel shows pPIPAGUS containing the *ssb_Xoc_* promoter and an imperfect PIP-box (TTCGC-N_19_-TTCGT) fused with a promoter-less *gusA* gene. Lower panel shows pPIPBGUS with a mutated *ssb_Xoc_* promoter (the first TT nucleotides replaced with AA) fused with *gusA*. (B) â-glucuronidase (GUS) activity in the *hrp*-inducing medium XOM3. Plasmids pPIPAGUS and pPIPBGUS were transferred into the wild-type RS105 and mutants RΔ*hrpG* and RΔ*hrpX*. The recombinant strains were then grown in *hrp*-inducing medium XOM3 for 16 h. GUS activity was determined by measuring the OD at 415 nm using ρ-nitrophenyl-â-D-glucuronide as a substrate. Data represent the mean ± SD of triplicate measurements. The different letters above each horizontal column indicate significant differences at *P = *0.01 (*t* test). (C) Expression of *ssb_Xoc_* in *hrp*-inducing and nutrient-rich media. Real-time quantitative RT-PCR was used to compare relative expression of *ssb_Xoc_* in *X. oryzae* pv. *oryzicola* strains RS105, RΔ*hrpG*, and RΔ*hrpX*. RNA was isolated from strains grown in a nutrient-rich medium (NB) and the *hrp*-inducing medium (XOM3) for 16 h. The relative mRNA levels of *ssb_Xoc_* in the *hrpG* and *hrpX* mutants were calculated with respect to the wild-type strain. Values given are the means ± SD of triplicate measurements from a representative experiment, and similar results were obtained in two other experiments. Different letters above horizontal columns represent significant differences at *P = *0.01 using the Student’s *t* test. (D) Real-time RT-PCR evaluation of *ssb_X_* expression in *Xanthomonas* species. Strains were grown at 28°C for 16 h in NB or one of the following *hrp*-inducing media: XOM3 for *X. oryzae* pv. *oryzicola* RS105 and *X. oryzae* pv. *oryzae* PXO99^A^ (Xiao et al. 2007), XVM2 for *X. axonopodis* pv. *citri* 306 & *X. campestris* pv. *vesicatoria* 85-10, and MMX for *X. campestris* pv. *campestris* 8004 (see methods). Relative mRNA quantitative of *ssb_X_* was calculated with respect to the levels observed for wild-type strains grown in NB. Genes encoding 16S rRNA were used as internal controls. Data represent means ± SD of triplicate measurements (P = 0.01, *t* test).

The down-regulated expression of *ssb_Xoc_* in the RΔ*hrpX* and RΔ*hrpG* mutants indicates that *ssb_Xoc_* is positively regulated by HrpX and HrpG (Fig. 1). To investigate this further, we used promoter prediction software (HUhttp://www.fruitfly.org/seq_tools/promoter.html) to analyze the *ssb_Xoc_* promoter region in the *X. oryzae* pv. *oryzicola* BLS256 genome [61]. This analysis revealed an imperfect PIP-box (TTCGC-N_19_-TTCGT) upstream of the *ssb_Xoc_* start codon (Fig. 7A), suggesting that *ssb_Xoc_* may be regulated by HrpX [79]. To determine whether *ssb_Xoc_* expression depends on the putative PIP-box and HrpX, we constructed a recombinant plasmid pPIPAGUS, which contains the *ssb_Xoc_* promoter region fused to a promoter-less *gusA* in pUFR034, resulting in pPIPAGUS (Fig. 7A, Table S1). A mutated *ssb_Xoc_* promoter (first two TT nucleotides replaced with AA, see Fig. 7A) was also fused to *gusA* in pUFR034, generating pPIPBGUS (Fig. 7A, Table S1). Plasmids pPIPAGUS and pPIPBGUS were transformed into the wild-type RS105 and mutants RΔ*hrpX* and RΔ*hrpG*, incubated in the *hrp*-inducing medium XOM3, and GUS activities were measured. GUS activity of pPIPAGUS was significantly lower in the *hrpG* and *hrpX* mutants than in the wild-type strain (*P = *0.01, *t* test). GUS activity of the mutated *ssb_Xoc_* transcriptional fusion (pPIPBGUS) was similar in the wild-type, RΔ*hrpG*, and RΔ*hrpX* strains (*P = *0.01, *t* test) (Fig. 7B). We also used real-time PCR to evaluate expression levels of *ssb_Xoc_* in strains RS105, RΔ*hrpG*, and RΔ*hrpX*. The expression of *ssb_Xoc_* was higher in the *hrp*-inducing medium XOM3, but significantly reduced in the nutrient-rich NB, regardless of the genetic background (Fig. 7C). *ssb_Xoc_* expression levels were consistently lower in RΔ*hrpG* and RΔ*hrpX* than the wild-type (Fig. 7C). Collectively, results indicate that *ssb_X_* transcription is positively regulated by HrpG and HrpX and suggest that *ssb_Xoc_* contains a PIP-box that is likely regulated by HrpX.

Our results suggest that the HR-eliciting SSB_X_ protein is highly conserved in *Xanthomonas* species (Fig. S1, Fig. 2C), leading us to investigate whether PIP-box promoters drive *ssb_X_* expression in other xanthomonads or not. Interestingly, PIP-box promoters were identified upstream of the *ssb* coding sequences in *X. oryzae* pv. *oryzae* PXO99^A^, *X. campestris* pv. *vesicatoria* 85-10, *X. axonopodis* pv. *citri* 306, and *X. campestris* pv. *campestris* 8004 (data not shown), implying that *ssb* expression in these bacteria is also induced *in planta*. Thus, we performed real-time PCR to investigate the expression levels of *ssb_X_* in various *Xanthomonas* species grown in the *hrp*-inducing media (see Methods). *ssb_X_* expression was significantly higher in *hrp*-inducing media than in the nutrient-rich media, which suggests that these genes have a functional PIP-box and are regulated by HrpX.

## Discussion

In this report, we demonstrate that single-stranded DNA-binding proteins from *Xanthomonas* elicit HR in tobacco. This activity was not demonstrated with SSB proteins obtained from other prokayrotes, so it may be a unique feature of *Xanthomonas*. Like Hpa1, SSB_Xoc_ contributes both to bacterial growth and virulence in rice (Fig. 6) and also triggers programmed cell death (Fig. 2, 3). This is the first report that *Xanthomonas* produce a highly-conserved SSB_X_ protein that functions as a harpin-like protein. Furthermore, we showed that SSB_X_ binds nonspecifically to single-stranded DNAs (Fig. S3), perhaps a potential role in protecting ssDNA from nucleases [62]. Unlike some PAMPs with a very narrow distribution, such as Ax21 in *X. oryzae pv. oryzae* [80], SSBx may be widely distributed in *Xanthomonas* species. It has been proposed that PAMPs are conserved throughout classes or genera of microbes and contribute to general microbial fitness [81].

Harpins are generally highly constrained structures that are difficult for plant pathogenic bacteria to alter because they have evolved to help bacteria avoid recognition in plants. The first identified harpin, HrpN from *E. amylovora* [7], has been identified in related pathogens including *Pantoea stewartii* subsp. *stewartii* [82] and *D. dadantii* [83]. Another harpin, HrpZ, first identified in *P. syringae* pathovars [6], [84], but was later shown to be present in nonpathogenic pseudomonads including *P. putida* and *P. fluorescens* [85]. The harpin HrpW, which contains harpin and pectate-lyase domains, is widely conserved across genera and has been identified in *E. amylovora* [41], *P. syringae* [42], *D. dadantii* [86], *R. solanacearum* [39], and *X. campestris* pv. *campestris* [46]. Interestingly, there is no HrpW homologue in the genomes of *X. oryzae pv. oryzae*, *X. oryzae* pv. *oryzicola*, *X. campestris* pv. *vesicatoria*, or *X axonopodis* pv. *citri* [46], [61], [87]. Thus, some harpins may have a more narrow distribution. For example, *R. solanacearum* contains an SSB protein but this does not elicit HR in tobacco (Fig. 2D), possibly because SSB_Rs_ lacks the conserved glycine-rich region of SSB_X_ (Fig. S1) and the pathogen causes bacterial wilt in tobacco. It is also interesting to recall that flg15, a truncated version of flagellin-derived flg22, does not act as an elicitor in *Arabidopsis* or *N. benthamiana*, while it is fully active in tomato [88]. In *Xanthomonas*, only Hpa1 [8], [43] and SSB_X_ (Fig. 2; this study) have been identified as harpins that elicit HRs in tobacco. However, the *hpa1-ssb_Xoc_* double mutant RΔ*hpa1*Δ*ssb_X_* still elicited HR in the wild-type and SA-deficient (*NahG*) tobacco plants (data not all shown), implying that *Xanthomonas* produce other elicitors that trigger a SA-independent HR. This double mutant is a valuable resource for identifying other harpin(s) that exist in *Xanthomonas*.

Mutations in the T3SS of Gram-negative phytopathogenic bacteria often disrupt the ability of the pathogen to elicit HR in tobacco, presumably because harpin proteins are secreted via the T3SS [6]–[8], [41]–[43]. In Hrp group II, the *cis*-acting PIP-box promoter is followed by a −10 box, and collectively these elements indicate regulation via HrpX [76], [79], [89]. However, the PIP-box in *ssb_X_* genes is not followed by the −10 box, possibly because proteins in addition to HrpX are involved in regulation [44], [60]. At the amino acid level, harpin proteins possess T3SS signals that are characterized by at least 20% Pro and Ser residues in the first 50 amino acids at the N-terminus; this protein signature is required for secretion through the T3SS [78], [90]. Intriguingly, SSB_X_ protein does not have the T3SS signal (Fig. S1), but is secreted, as Hpa1, through the T3SS independently of HpaB and HpaP (Fig. 6D, 6E). Thus, the compelling topics for future studies include understanding of how *ssb_X_* is regulated by HrpX and the mechanism of SSB_x_ secretion.

Hpa1 and SSB_X_ function for HR induction in tobacco (Fig. 2) and are required for full virulence in rice (Fig. 6); these dual functions are difficult to reconcile. Harpins may activate defense by entering into plant membranes and modulating ion channels or may be recognized by unidentified receptor(s) [24]. Recent reports show that the key α-helical domain in harpins, including HpaG of *X. campestris pv. glycines* that is orthologous to Hpa1, is required for amyloidogenesis [23], [27]. However, the Hpa1 α-helix does not show any similarity to SSB_X_ proteins (data not shown). We speculate that the conserved domain of SSB_X_ proteins (Fig. S1) is required for HR induction, and experiments to investigate this hypothesis are underway in our lab. The fact that harpins do not elicit HR in host plants suggests that the recognition of harpins differs in host and non-host plants. It is also important to mention that harpins function in the translocation of T3SEs, and this function is required for a full level of virulence in host plants [91]. Whether SSB_X_ plays a role in T3SE translocation or not, remains unknown; however, the mutation in ssb_Xoc_ did not impaired the secretion of Hpa1 (Fig. 6E) and the *ssb_Xoc_*-*hpa1* double mutant still caused disease in rice (Fig. 6A, B, C), so at least some effectors were delivered to rice cells inoculated with the double mutant (Fig. 6). These findings are reminiscent of those reported for *P. syringae* pv. *tabaci* flagellin mutants, which were abrogated in elicitor activity and displayed reduced virulence due to impaired motility [92], [93].

Plant immune responses triggered by harpins are often associated with HR and SAR [6], [7], [24], [28]. In the present study, we show that SSB_x_ induces PCD (Fig. 2E), the oxidative burst (Fig. 3C), the expression of HR and SAR marker genes (Fig. 2F and 3F), and callose deposition (Fig. 3D), which stimulate plant defense. SSB_x_-induced HR, like Hpa1-induced, could be blocked by eukaryotic metabolic *inhibitors (* *Fig. 2D* *)*. It will be interesting to determine whether SSB proteins from diverse genera can elicit HR, and such studies will help us understand how pathogens recruit molecules that are instrumental for bacterial fitness and re-deploy them as agents for plant defense.

Although plant-associated microbes can potentially produce many molecules with conserved signatures, only a few PAMPs have been identified, and most of these trigger a similar set of responses. In the current study, we evaluated the expression of PTI signature genes, e.g. *BAK1*, *BIK1* and *MAP3K* [19], [73], [94]. The activation of these genes by Hpa1 and SSB_Xoc_ (Fig. 3) further supports the contention that PTI is a variant of ETI [18], [81]. Recently, Ax21 of *X. oryzae* pv. *oryzae*, which is perceived by Xa21 in rice [80], was shown to be recognized by FLS2 in *Arabidopsis* [95]. Thus it remains possible that Hpa1 and SSB_Xoc_, like Ax21, may recruit FLS2 in a receptor complex together with other receptors and adaptors that modulate PTI. The identity of receptors for Hpa1 and SSB_Xoc_ and whether these PAMPs interact with FLS2 remains unclear.

There is abundant evidence in the literature showing that harpins display pleiotropic effects both on HR & SAR and also impact plant growth [28], [29], [96]. Our results also showed that Hpa1 and SSB_Xoc_ enhanced growth of *Arabidopsis* and tobacco (Fig. 5); this was correlated with increased expression of *EIN2* and *PR4* genes (Fig. 3F) that are essential for Eth-signaling [75]. This is consistent with the contention that Eth-signaling regulates the accumulation of the FLS2 receptor and is required for the oxidative burst leading to PTI [97]; thus, Hpa1- and SSB_Xoc_-mediated plant immunity may also require Eth-signaling. Eth- and SA- signaling may be regulated by WRKY transcription factors that are phosphorylated by the MAPK cascade [98], [99]. Nevertheless, SSB_x_ may possibly have pleiotropic effects in plants.

**Figure 8:**
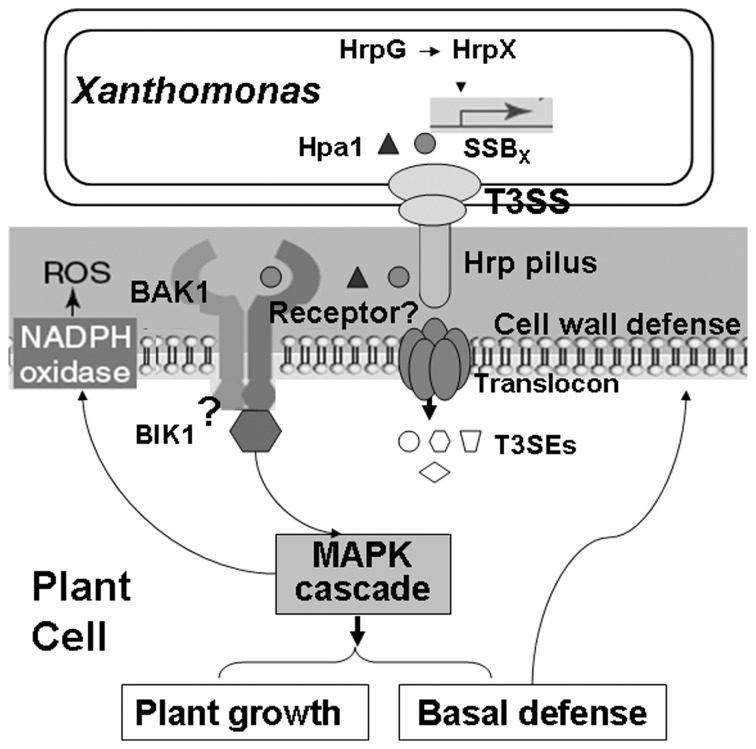
Working model of SSB_X_ function. SSB_Xoc_, like Hpa1, which are regulated at the transcriptional level by HrpG and HrpX, are potentially secreted into the plant apoplast via the T3SS. The grey circle and black triangle indicate SSB_X_ and Hpa1 proteins that may be secreted through the Hrp pilus (encoded by *hrpE*), but not translocated through the translocon as other T3SEs (different white shapes), and then are possibly recognized by an unidentified receptor (question mark) which associates with *BAK1*. This interaction may result in phosphorylation of *BIK1* and subsequent phosphotransfer to the MAPK cascade to activate the expression of genes involving in SA-, JA- and Eth-signaling pathways that lead to induced resistance (SAR and/or ISR) accompanied by callose deposition on cell walls and enhanced plant growth. MAPK signaling regulates NADPH oxidase-dependent oxidative burst in the early stages of plant defense.

Based on the results of this study, we propose a working model for SSB_X_ function that is also applicable to Hpa1 (Fig. 8). The *ssb_x_* gene in *Xanthomonas* is regulated by HrpG and HrpX; the latter protein potentially binds to the PIP-box promoter and activates transcription. Although the mechanistic basis of secretion is not totally understood, SSB_x_ secretion depends on the functional T3SS, but does not depend on the presence of HpaB and HpaP. We hypothesize that, besides protecting ssDNAs from nucleases in bacterial cells, SSB_x_, possibly like Hpa1, is secreted through the T3SS, but not translocated into plant cells, and perceived in plant apoplast where it is recognized by an unknown receptor, possibly a plasma membrane-localized PAMP receptor-like kinase (RLKs) that recruits other proteins, like BAK1, and activates downstream signal transduction cascades for HR induction (Fig. 8). We speculate that signaling leads to expression of Eth-dependent genes for plant growth and SA- or JA-dependent genes for plant defense. These hypotheses are the subject of ongoing experiments in our laboratory by undertaking the investigation of an unknown SSB_X_-interacting protein in plants.

## Supporting Information

Figure S1 **Comparison of single-stranded DNA-binding proteins in** ***Xanthomonas*** **species and other prokaryotes by multiple sequence alignment.** The sequences within the black dashed-line rectangle represent conserved region in *Xanthomonas* but variable in other prokaryotes. Protein accession numbers are indicated. The abbreviations are as follows: *Xoc*, *X. oryzae* pv. *oryzicola*; *Xoo*, *X. oryzae* pv. *oryzae*; *Xcv*, *X. campestris* pv. *vesicatoria*; *Xcc*, *X. campestris* pv. *campestris*; *Rs*, *Ralstonia solanacearum*; *Ya*, *Yersinia aldovae*; *Ea*, *Erwinia amylovora*; *Pst*, *Pseudomonas syringae* pv. *tomato*; *Ec*, *Eschericha coli*, and *Xf*, *Xylella fastidiosa*.
(TIFF)

Figure S2 **Phylogenetic analysis of SSB proteins in various bacterial species.** A neighbor-joining bootstrap tree was derived from the amino acid sequences of SSB proteins using the Vector NTI Align program (http://www.invitrogen.com). Protein accession numbers are indicated after the bacterial species or strain designation. Based on phylogenetic analysis, SSB proteins were classified into one of three groups (I, II and III) for HR induction in nohost tobacco.
(TIFF)

Figure S3 **SSB_Xoc_ binds to single-stranded DNAs in electrophoretic mobility shift assays (EMSA).** Randomly synthesized DNA1 and DNA2 (Table S2) were labeled with the Biotin 3′ End DNA Labeling Kit (Thermo, USA). EMSA was performed using protocols supplied with the LightShift Chemoluminescent EMSA Kit (Thermo, USA). Five µg of purified SSB_Xoc_ protein was mixed with 20 µl of the binding buffer and 20 fmol of biotin-labeled DNA1 (left panel) or DNA2 (right panel); in competition assays (lanes marked with*), labeled DNA was mixed with a 200-fold molar excess of unlabeled DNA1 or DNA2. The mixtures were incubated at room temperature for 20 min. Samples were then loaded on 5% polyacrylamide gels in 0.5X TBE buffer (pH 8.3). Gels were transferred to Hybond N+ membranes (Amersham, Pharmacia), and signals were detected by chemoluminescence according to the manufacturer’s instructions. The experiment was repeated twice and similar results were obtained. Lanes that are labeled (−) do not contain SSB_x_; lanes labeled (+) contain SSB_Xoc_ and DNA. The middle lane in each panel clearly shows the retardation of DNA mobility due to SSB_Xoc_ binding.
(TIFF)

Table S1 **Strains and plasmids used in this study.**
(DOC)

Table S2 **Primers used in this study.**
(DOC)

Table S3 **Amino acid identity between SSB_X_ in** ***X. oryzae*** **pv.** ***oryzicola*** **RS105 and homologues in other bacteria.**
(DOC)
