# Supplementary figures and images for: A Highly-Conserved Single-Stranded DNA-Binding Protein in Xanthomonas Functions as a Harpin-Like Protein to Trigger Plant Immunity
Source: PLoS One. 2013 Feb 13;8(2):e56240. doi: 10.1371/journal.pone.0056240 (PMC3571957; doi:10.1371/journal.pone.0056240)

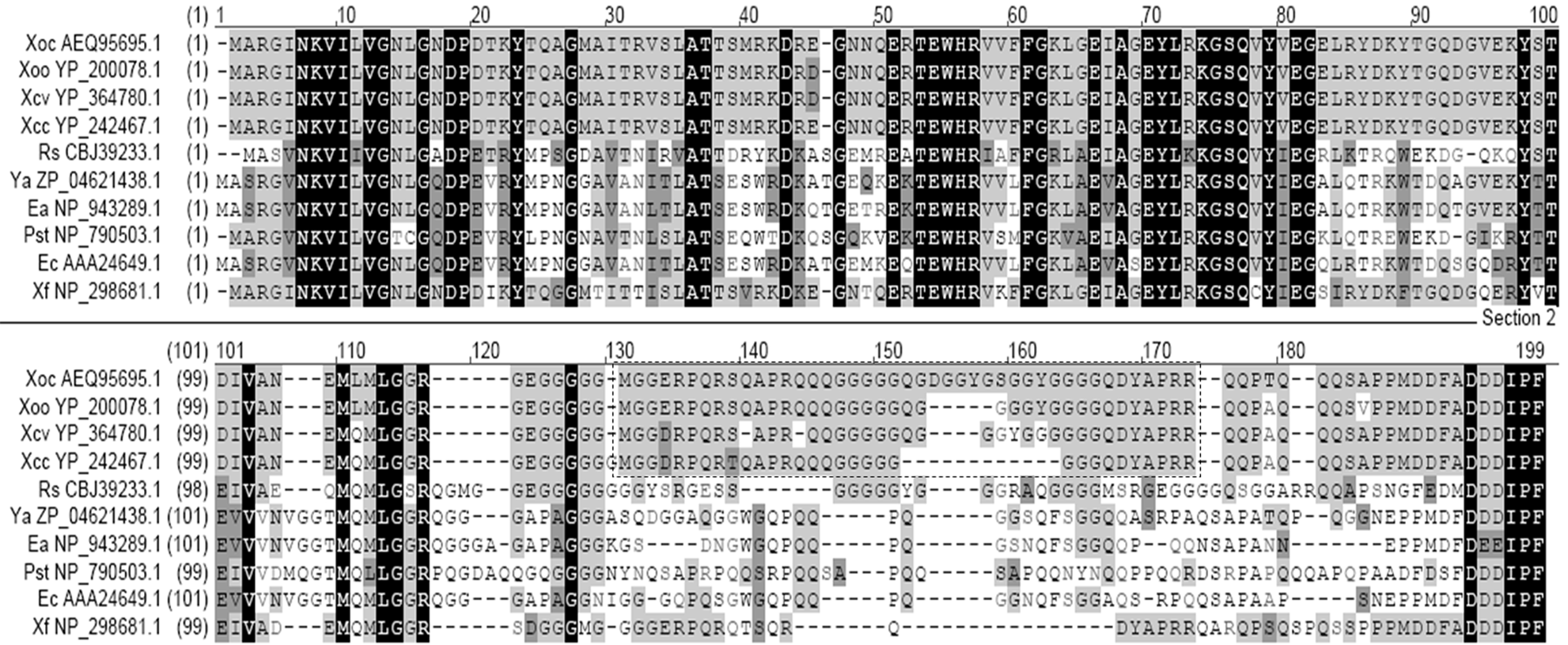

Supplement: Figure S1 — Comparison of single-stranded DNA-binding proteins in Xanthomonas species and other prokaryotes by multiple sequence alignment. The sequences within the black dashed-line rectangle represent conserved region in Xanthomonas but variable in other prokaryotes. Protein accession numbers are indicated. The abbreviations are as follows: Xoc, X. oryzae pv. oryzicola; Xoo, X. oryzae pv. oryzae; Xcv, X. campestris pv. vesicatoria; Xcc, X. campestris pv. campestris; Rs, Ralstonia solanacearum; Ya, Yersinia aldovae; Ea, Erwinia amylovora; Pst, Pseudomonas syringae pv. tomato; Ec, Eschericha coli, and Xf, Xylella fastidiosa. (TIFF) [file pone.0056240.s001.tiff]

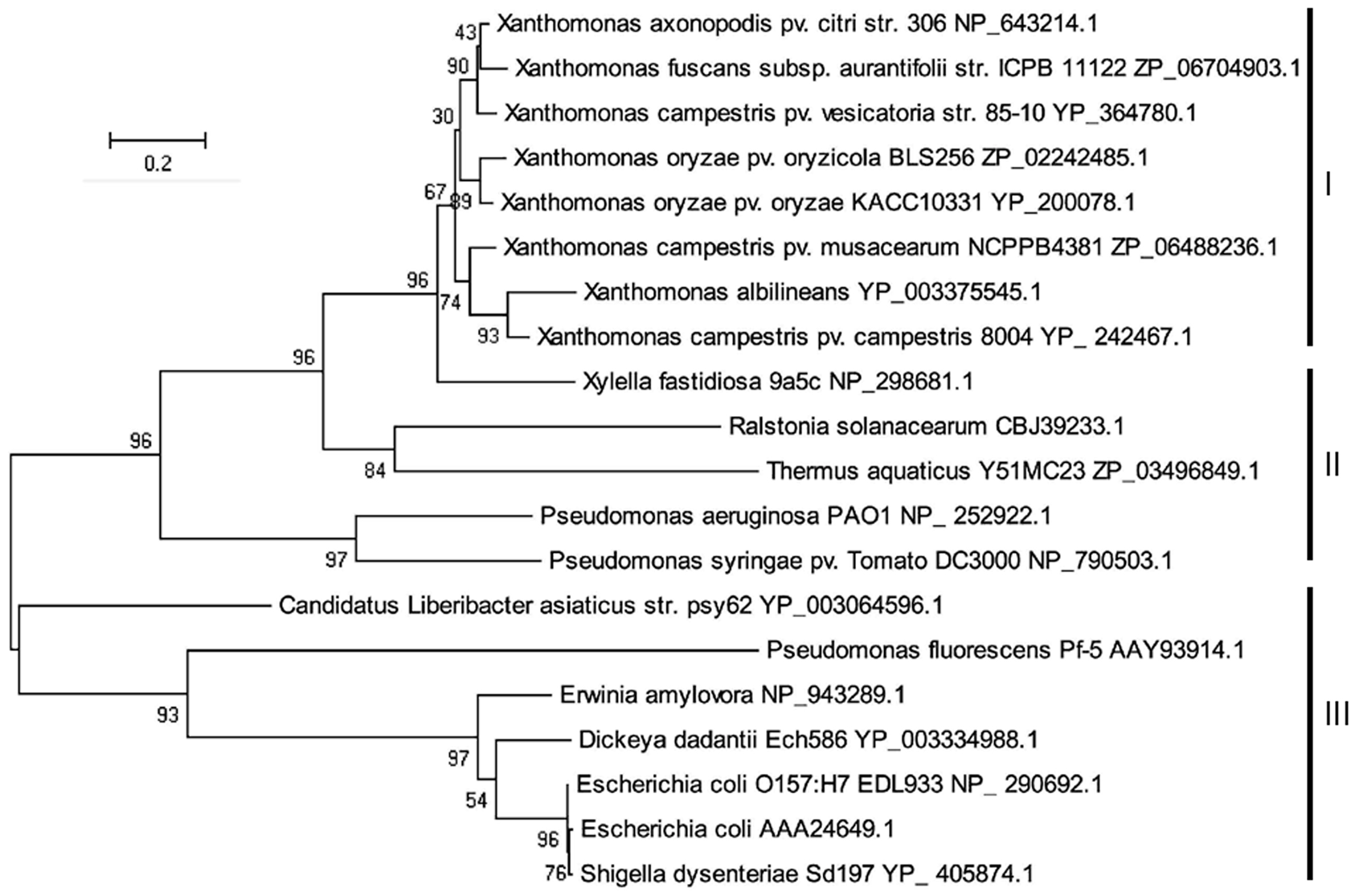

Supplement: Figure S2 — Phylogenetic analysis of SSB proteins in various bacterial species. A neighbor-joining bootstrap tree was derived from the amino acid sequences of SSB proteins using the Vector NTI Align program (http://www.invitrogen.com). Protein accession numbers are indicated after the bacterial species or strain designation. Based on phylogenetic analysis, SSB proteins were classified into one of three groups (I, II and III) for HR induction in nohost tobacco. (TIFF) [file pone.0056240.s002.tiff]

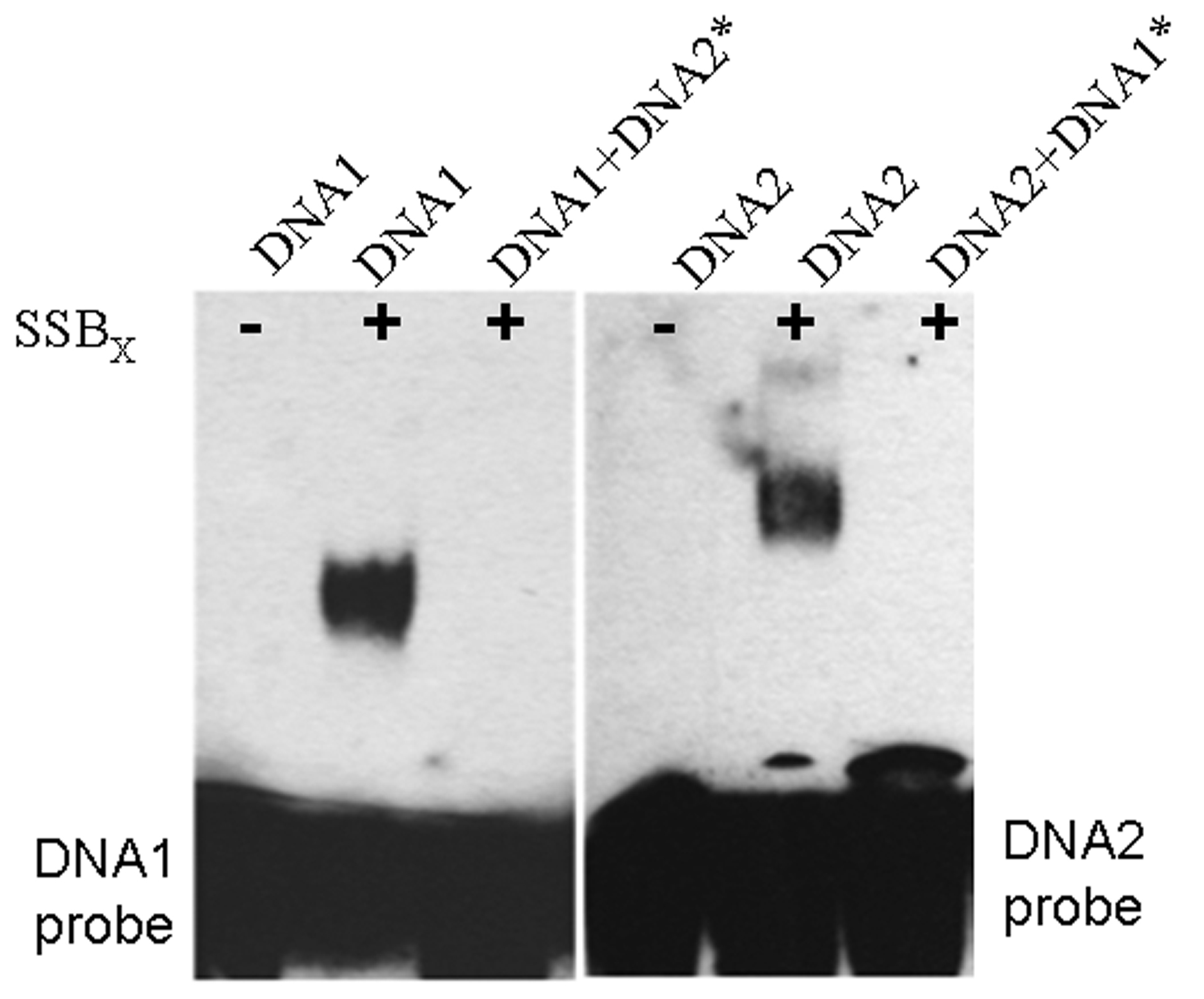

Supplement: Figure S3 — SSBXoc binds to single-stranded DNAs in electrophoretic mobility shift assays (EMSA). Randomly synthesized DNA1 and DNA2 (Table S2) were labeled with the Biotin 3′ End DNA Labeling Kit (Thermo, USA). EMSA was performed using protocols supplied with the LightShift Chemoluminescent EMSA Kit (Thermo, USA). Five µg of purified SSBXoc protein was mixed with 20 µl of the binding buffer and 20 fmol of biotin-labeled DNA1 (left panel) or DNA2 (right panel); in competition assays (lanes marked with*), labeled DNA was mixed with a 200-fold molar excess of unlabeled DNA1 or DNA2. The mixtures were incubated at room temperature for 20 min. Samples were then loaded on 5% polyacrylamide gels in 0.5X TBE buffer (pH 8.3). Gels were transferred to Hybond N+ membranes (Amersham, Pharmacia), and signals were detected by chemoluminescence according to the manufacturer’s instructions. The experiment was repeated twice and similar results were obtained. Lanes that are labeled (−) do not contain SSBx; lanes labeled (+) contain SSBXoc and DNA. The middle lane in each panel clearly shows the retardation of DNA mobility due to SSBXoc binding. (TIFF) [file pone.0056240.s003.tiff]
