# Supplementary material for: A Highly-Conserved Single-Stranded DNA-Binding Protein in Xanthomonas Functions as a Harpin-Like Protein to Trigger Plant Immunity
Source: PLoS One. 2013 Feb 13;8(2):e56240. doi: 10.1371/journal.pone.0056240 (PMC3571957; doi:10.1371/journal.pone.0056240)
Supplement: Table S1 — Strains and plasmids used in this study. (DOC) [file pone.0056240.s004.doc]

Table S1. Strains and plasmids used in this study

| **Strain** | **Properties** | **Source** |
| --- | --- | --- |
| *Ec* DH5α | *recA1 hsdR17*(r_K_^—^ m_K_^+^) *phoA supE44 λ^—^ thi-l gyrA96 relA1* | Clontech |
| *Ec* BL21(DE3) | F^—^ *ompT hsdS20*(r_b_^—^ m_b_^—^) *gal* | Novagen |
| *Xoc* RS105 | Wild-type, Rif^r^, Chinese race 2 | This lab |
| *Xoc* RΔ*hrpX* | *hrpX* deletion mutant of strain RS105; Rif^R^ | [44] |
| *Xoc* RΔ*hrpG* | *hrpG* deletion mutant of strain RS105; Rif^R^ | [44] |
| *Xoc* RΔ*hrcV* | *hrcV* deletion mutant of strain RS105; Rif^R^ | [76] |
| *Xoc* RΔ*hrcC* | *hrcC* deletion mutant of strain RS105; Rif^R^ | [44] |
| *Xoc* RΔ*hrpE* | *hrpE* deletion mutant of strain RS105; Rif^R^ | [76] |
| *Xoc* RΔ*hpaB* | *hpaB* deletion mutant of strain RS105; Rif^R^ | [57] |
| *Xoc* RΔ*hpaP* | *hpaP* deletion mutant of strain RS105; Rif^R^ | [44] |
| *Xoc* RΔ*hpa1* | *Hpa1* deletion mutant of strain RS105; Rif^R^ | [44] |
| *Xoc* RΔ*ssb_X_* | *ssb_X_* knock-out mutant of strain RS105; Rif^R^ | This study |
| *Xoc* RΔ*hpa1*Δ*ssb_X_* | *hpa1* *ssb_X_* deletion mutant of strain RS105; Rif^R^ | This study |
| *Xoc* CRΔ*ssb_X_* | RΔ*ssb_X_* containing pUssb; Rif^R^, Km^R^ | This study |
| *Xoo* PXO99^A^ | Wild-type, Philippine race 6, causal agent of bacterial blight of rice | Lab collection |
| *Xcc* 8004 | Wild-type, causal agent of black rot in crucifers | Lab collection |
| *Xac* 306 | Wild-type, causal agent of citrus canker | Lab collection |
| *Xcv* 85-10 | Wild-type, causes bacterial spot disease in tomato and pepper | Lab collection |
| *Ea* 0065 | Wild-type, causal agent of fire blight in apple and pear | Lab collection |
| *Rs* ZJ3721 | Wild-type, causal agent of bacterial wilt of tomato; Zhejiang, China | [40] |
| *Pf* Pf-55 | A biocontrol agent for plant diseases | Lab collection |
| *Pst* DC3000 | Wild-type, causes bacterial speck of tomato and Arabidopsis | Lab collection |
| *Agrobacterium tumefaciens G*V3101 | Contains disarmed Ti plasmid; Gm^R^, Rif^R^ | [45] |
| *Alternaria alternata* TBA28 | Wild-type, causal agent of tobacco brown spot | Lab collection |
| **Plasmid** |  |  |
| pMD18-T | pUC *ori*, cloning vector, Ap^R^ | TaKaRa |
| pUFR034 | *incW*, *mob^+^, lacZa^+^*, cosmid vector, Km^R^ | [58] |
| pKMS1 | Suicide vector derived from pK18mobGII, *sacB^+^*; Km^R^ | [57] |
| pET41a(+) | pBR322 origin, *lacI,* His-Tag, GST-Tag, S-Tag; Km^R^ | Novagen |
| pPIPAGUS | 300 bp, *ssb_X_* promoter fused with *gusA* in pUFR034; Km^R^ | This study |
| pPIPBGUS | 300 bp, *ssb_X_* promoter with mutant PIP-box fused to *gusA* in pUFR034; Km^R^ | This study |
| pUssb | Intact *ssb_X_* ORF plus *c-myc* under its own native promoter in pUFR034; Km^R^ | This study |
| pUhpa1 | A 676-bp fragment of *hpa1* with its own promoter fused with a c-Myc tag; Km^R^ | [44] |
| pKΔ*ssb_X_* | 1102 bp fusion upstream and downstream of *ssb_Xoc_* in pKMS1; Km^R^ | This study |
| pSSB*_Xoc_* | 552 bp *ssb_X_* of *Xoc* cloned in pET41a(+), GST-tagged; Km^R^ | This study |
| pSSB*_Xoo_* | 537 bp *ssb_Xoo_* of *Xoo* cloned in pET41a(+), GST-tagged; Km^R^ | This study |
| pSSB*_Xac_* | 543 bp *ssb_Xac_* of *Xac* cloned in pET41a(+), GST-tagged; Km^R^ | This study |
| pSSB*_Xcc_* | 519 bp *ssb_Xcc_* of *Xcc* *ssb_Xcc_* cloned in pET41a(+), GST-tagged; Km^R^ | This study |
| pSSB*_Xcv_* | 534 bp *ssb_Xcv_* of *Xcv* cloned in pET41a(+), GST-tagged; Km^R^ | This study |
| pSSB*_Ea_* | 525 bp *ssb_Ea_* of *Ea* cloned in pET41a(+), GST-tagged; Km^R^ | This study |
| pSSB*_Ec_* | 537 bp *ssb_Ec_* of *E. coli* cloned in pET41a(+), GST-tagged; Km^R^ | This study |
| pSSB*_Rs_* | 543 bp *ssb_Rs_* of *Rs* cloned in pET41a(+), GST-tagged; Km^R^ | This study |
| pSSB*_Pst_* | 570 bp *ssb_Pst_* of *Pst* cloned in pET41a(+), GST-tagged; Km^R^ | This study |
| pSSB*_Pf_* | 564 bp *ssB_Pf_* of *Pf* cloned in pET4aa(+), GST-tagged; Km^R^ | This study |
| pHpa1 | 414 bp *hpa1* of *Xoc* cloned in pET41a(+), GST-tagged; Km^R^ | [34] |
| pgR107 | PVX vector with *CaI*I-*Sma*I-*SaI*I sites; Km^R^ | [54] |
| pPVXbax | 174 bp *bax* cloned in pgR107; Km^R^ | [55] |
| pPVXhpa1 | 414 bp *hpa1* cloned in pgR107 at *CaI*I and *SaI*I sites; Km^R^ | This study |
| pPVXssb_X_ | 542 bp ssb_Xoc_ cloned in pgR107 at *CaI*I and *SaI*I sites; Km^R^ | This study |

Note: Rif^R^, Sp^R^, Km^R^, and Sp^R^ indicate resistant to rifampin, kanamycin, spectinomycin, and streptomycin, respectively;*Ea, Erwinia amylovora; Ec, Escherichia coli; Pst, Pseudomonas syringae* pv. *tomato; Rs, Ralstonia solanacearum; Xac, Xanthomonas axonopodis pv. ctiri; Xoc*, *Xanthomonas oryzae* pv. *oryzicola*; *Xoo*, *Xanthomonas oryzae* pv. *oryzae*; Xcc, *Xanthomonas campestris* pv. *campestris*; *Xcv*, *Xanthomonas campestris* pv. *vesicatoria*; *Pf*, *Pseudomonas fluorescens*.
