# Supplementary material for: A Highly-Conserved Single-Stranded DNA-Binding Protein in Xanthomonas Functions as a Harpin-Like Protein to Trigger Plant Immunity
Source: PLoS One. 2013 Feb 13;8(2):e56240. doi: 10.1371/journal.pone.0056240 (PMC3571957; doi:10.1371/journal.pone.0056240)
Supplement: Table S2 — Primers used in this study. (DOC) [file pone.0056240.s005.doc]

**Table S2. Primers used in this study.**

| **Primers** | **Sequence (5’→3’; restriction sites underlined)** | **Description of fragment amplified** |
| --- | --- | --- |
| *ssb*I-F  *ssb*I-R | TTGGATCCTGGGCACAACCGGTCTCTTC  ATTCTCGAGGTTGCCGACGAGGATGACT | 421 bp fragment flanking *ssb_X_* on left |
| *ssb*II-F  *ssb*II-R | CTCGAGACGACGATATCCCGTTCTGAGG  CTGCAGAGTTGCCGAGCTCGGTCTGATG | 692 bp fragment flanking *ssb_X_* on right |
| pssb-F  pssb-R | ATAGAATTCATCTGGTTGATCAGCACGA/ TATAAGCTTAGGTGCTTCCGAGATGATG | 408 bp promoter region upstream of *ssb_X_* |
| mpssb-F | ATAGAATTCATGCCAGGCGAACGTGATGT | 408 bp promoter region where TT is replaced with AA in the first motif of the PIP-box |
| ssbcmyc | ATAAAGCTTCTACAGATCTTCTTCAGAAATAAGTTTTTGTTCGAACGGGATATCGTCGTC | 828 bp fragment of *ssb_Xoc_* with its own promoter fused with a c-Myc tag, amplified with the primer pssb-F |
| *gusA*-F  *gusA*-R | ATAAAGCTTTTACGTCCTGTAGAAACCC  TAAGAATTCTCATTGTTTGCCTCCCTGCTG | 1831 bp *gusA* |
| *hpa1-F*  *hpa1-R* | AATGAATTCATGAACTCTTTGAAC  TAAGTCGACTTACTGCATCGATCC | 414 bp *hpa1* (AY875714 ) |
| *ssb_X_*-F  *ssb_X_*-R | TAGAATTCATGGCCCGCGG  ATTGTCGACTCAGAACGGGATATCGT | 552 bp *ssb_Xoc_* (AEQ95695.1) |
| *ssb_Xoo_*-F  *ssb_Xoo_*-R | TAGAATTCATGAGCCTGGCCACCACCAGCA  ATTGTCGACTCAGAACGGGATATCGT | 444 bp *ssb_Xoo_* (YP_200078.1) |
| *ssb_Xac_*-F  *ssb_Xac_*-R | TAGAATTCATGGCCCGCGG  ATTGTCGACTCAGAACGGGATATCGT | 543 bp *ssb_Xac_* (NP_643214.1) |
| *ssb_Xcv_*-F  *ssb_Xcv_*-R | TAGAATTCATGGCCCGCGG  ATTGTCGACTCAGAACGGGATATCGT | 534 bp *ssb_Xcv_* (YP_364780.1) |
| *ssb_Xcc_*-F  *ssb_Xcc_*-R | TAGAATTCATGGCCCGCGG  ATTGTCGACTCAGAACGGGATATCGT | 519 bp *ssb_Xcc_* (YP_242467.1) |
| *ssb_Ea_*-F  *ssb_Ea_*-R | TAAATCGATGAATTCATGGCTTCAAGAGGCGTT  AAAGTCGACTCAAAATGGGATCTCTTCGTCAAA | 525 bp *ssb_Ea_* (NP_943289.1) |
| *ssb_Ec_*-F  *ssb_Ec_*-R | AAGAATTCATGGCCAGCAGAGGCGTAAACAAG  TTGTCGACAATCAGAACGGAATGTCATCATCA | 537 bp *ssb_Ec_* (AAA24649.1) |
| *ssb_Rs_*-F  *ssb_Rs_*-R | TTGAATTCATGGCGTCCGTCAACAAAGTCATC  AAGTCGACTTAGAAAGGAATATCGTCATCCAT | 543 bp *ssb_Rs_* (CBJ39233.1) |
| *ssb_Pst_-*F  *ssb_Pst_*-R | GAATTCATGGCCCGTGGGGTTAACAAAGTC  AAGCTTTTAGAACGGAATATCGTCATCAAA | 570 bp *ssb_Pst_* (NP_790503.1) |
| *16s RNA-F*  *16s RNA-R* | CAGCCACACTGGAACTGAGA  GTTAGCCGGTGCTTCTTCTG | Amplifies 16s RNA (JA074430.1 and JA074431.1) |
| *BAK1-*F  *BAK1-*R | GAATTGGACTTGTCTTTCAATAATTTCTCA  GGATAAGATCTTTCAAACTTTGACAATGACC | 500 bp of *BAK1* (NM_001203975.1); used as probe in northern blot |
| *BIK1-F*  *BIK1-F* | CTTTGGTTGTGTCTTTAAAGG  TGCATTCAAGTGACCTGATGA | 500 bp of *BIK1* (NM_129522.4); used as probe in northern blot |
| *NPR1-F*  *NPR1-R* | GATGTGTGTGTTTGTGTGGACAACGAGT  CCATCGGATGTCAGATCAGAAGGTCTAG | 500 bp of *NPR1* (NM_105369.2); used as probe for northern blot |
| *RAR1-F*  *RAR1-R* | TTCATGATGGGATGAAGAAGT  AACTGCAAGCAGTGTCATGAT | 500 bp of *RAR1* (HQ536957.1); used as probe in northern blot |
| *EIN2-F*  *EIN2-R* | CGCTATTCCAGTATGCGGATT  CCAGGGAATTATACCATTGAG | 500 bp of *EIN2* (HQ693477.1); used as probe in northern blot |
| *COl1-F*  *COI1-R* | GGATTGACTGATTTGGCGAAGG  TCCCTCACTGGCTACAACTCGT | 500 bp of *COI1* (NM_129552.3); used as probe in northern blot |
| *PR4-F*  *PR4-R* | AATTCGGCACGAGAAACCCTGGAAGA  CACAAGACACATAAACAAGAGTTATT | 500 bp of *PR4* (NM_111344.5); used as probe in northern blot |
| *MAP3K-*F  *MAP3K-*R | GAAATCATTTTACTTAGTAACTTGTCACAT  AACATGAGTAACTTTTGTTGTACTTTGGTT | 500 bp of *MAPKKK* (NM_116919.3); used as probe in northern blot |
| *HIN1-*F  *HIN1-*R | GAACGGAGCCTATTATGGCCCTTCC  CATGTATATCAATGAACACTAAACGCCGG | 500 bp of *HIN1* (NM_111997.2); used as probe in northern blot |
| *HSR203J-*F  *HSR203J-*R | TTGAACACACAATTCGGCGG  TTACTGCATCGATGCGCTGTC | 500 bp of *HSR203J* (AF212184.1); used as probe in northern blot |
| *PR1a-*F  *PR1a-*R | GGCGTTCTCTTTTCACAATTGCCTTCAT  AACGGACTTTCGCCTCTATAATTACCTG | 500 bp of *PR1a* (X06362.1); used as probe in northern blot |
| *PR1b-*F  *PR1b-*R | AGACCACCAAGTACTACTGCAC  CCACCAATCTTGTACACATCC | 500 bp of *PR1b* (X17680.1); used as probe in northern blot |
| DNA1  DNA2 | TTCGCGCGTACAAGCACAATTTCGCG AATAGCCCATCATCACCAGAAG | Synthesized for EMSA |
