# Supplementary material for: A Highly-Conserved Single-Stranded DNA-Binding Protein in Xanthomonas Functions as a Harpin-Like Protein to Trigger Plant Immunity
Source: PLoS One. 2013 Feb 13;8(2):e56240. doi: 10.1371/journal.pone.0056240 (PMC3571957; doi:10.1371/journal.pone.0056240)
Supplement: Table S3 — Amino acid identity between SSBX in X. oryzae pv. oryzicola RS105 and homologues in other bacteria. (DOC) [file pone.0056240.s006.doc]

Table S3. Amino acid identity between SSB_X_ in *X. oryzae* pv. *oryzicola* RS105 and SSB homologues in other bacteria

| **Accession No.** | **Species** | **Identities** | **Gaps** | **E-value** | | **%Gly** | |  |
| --- | --- | --- | --- | --- | --- | --- | --- | --- |
| AEQ95695.1 | *X. oryzae* pv. *oryzicola* BLS256 | 183/183 | 0/183 | | 2e-99 | | 20.22 | |
| YP_001914890.1 | *X. oryzae* pv. *oryzae* PXO99A | 174/183 | 5/183 | | 1e-63 | | 19.66 | |
| ZP_06704903.1 | *X. fuscans* subsp. *aurantifolii* ICPB 11122 | 168/183 | 7/183 | | 2e-61 | | 20.00 | |
| YP_364780.1 | *X. campestris* pv. *vesicatoria*  85-10 | 167/183 | 6/183 | | 2e-61 | | 20.34 | |
| ZP_06486176.1 | *X. campestris* pv. *vasculorum* NCPPB702 | 165/185 | 6/185 | | 4e-62 | | 20.99 | |
| ZP_06488236.1 | *X. campestris* pv. *musacearum* NCPPB4381 | 166/185 | 6/185 | | 4e-62 | | 21.55 | |
| YP_242467.1 | *X. campestris* pv. *campestris* 8004 | 162/184 | 13/184 | | 4e-62 | | 18.02 | |
| NP_643214.1 | *X. axonopodis* pv. *citri*  306 | 169/183 | 3/183 | | 2e-61 | | 21.67 | |
| YP_003375545.1 | *X. albilineans* | 143/185 | 16/184 | | 2e-67 | | 15.20 | |
| NP_298681.1 | *Xylella fastidiosa* 9a5c | 126/187 | 27/187 | | 2e-54 | | 13.41 | |
| ZP_03496849.1 | *Thermus aquaticus* Y51MC23 | 48/129 | 5/129 | | 3e-19 | | 10.49 | |
| YP_405874.1 | *Shigella dysenteriae* Sd197 | 89/186 | 14/186 | | 3e-33 | | 15.20 | |
| CBJ39233.1 | *Ralstonia solanacearum* | 61/109 | 2/109 | | 2e-34 | | 23.33 | |
| NP_790503.1 | *Pseudomonas syringae* pv. *tomato* DC3000 | 70/140 | 6/140 | | 6e-34 | | 10.05 | |
| AAY93914.1 | *Pseudomonas fluorescens* Pf-5 | 44/187 | 36/187 | | 2e-06 | | 9.52 | |
| NP_252922.1 | *Pseudomonas aeruginosa* PAO1 | 74/118 | 4/118 | | 8e-34 | | 9.70 | |
| NP_290692.1 | *Escherichia coli* O157:H7 EDL933 | 89/186 | 14/186 | | 2e-33 | | 16.29 | |
| AAA24649.1 | *Escherichia coli* | 89/189 | 20/189 | | 1e-34 | | 15.73 | |
| NP_943289.1 | *Erwinia amylovora* | 91/191 | 28/191 | | 5e-38 | | 16.09 | |
| YP_003334988.1 | *Dickeya dadantii* Ech586 | 86/186 | 10/186 | | 3e-30 | | 16.58 | |
| YP_003064596.1 | *Candidatus liberibacter asiaticus str.* psy62 | 66/185 | 28/185 | | 2e-30 | | 5.66 | |
